# Adrenal Incidentaloma

**DOI:** 10.1210/endrev/bnaa008

**Published:** 2020-04-08

**Authors:** Mark Sherlock, Andrew Scarsbrook, Afroze Abbas, Sheila Fraser, Padiporn Limumpornpetch, Rosemary Dineen, Paul M Stewart

**Affiliations:** 1 Department of Endocrinology, Beaumont Hospital, Dublin, Ireland; 2 Royal College of Surgeons in Ireland, Dublin, Ireland; 3 Department of Radiology, Leeds Teaching Hospitals NHS Trust, St James University Hospital, Leeds, UK; 4 Department of Endocrinology, Leeds Teaching Hospitals NHS Trust, St James University Hospital, Leeds, UK; 5 Department of Endocrine Surgery, Leeds Teaching Hospitals NHS Trust, St James University Hospital, Leeds, UK; 6 Faculty of Medicine & Health, University of Leeds, Worsley Building, Clarendon Way, Leeds, UK

**Keywords:** adrenal adenoma, adrenal incidentaloma, adrenal computed tomography, autonomous cortisol secretion, adrenal cortical carcinoma

## Abstract

An adrenal incidentaloma is now established as a common endocrine diagnosis that requires a multidisciplinary approach for effective management. The majority of patients can be reassured and discharged, but a personalized approach based upon image analysis, endocrine workup, and clinical symptoms and signs are required in every case. Adrenocortical carcinoma remains a real concern but is restricted to <2% of all cases. Functional adrenal incidentaloma lesions are commoner (but still probably <10% of total) and the greatest challenge remains the diagnosis and optimum management of autonomous cortisol secretion. Modern-day surgery has improved outcomes and novel radiological and urinary biomarkers will improve early detection and patient stratification in future years to come.

## Graphical Abstract

**Graphical Abstract:**
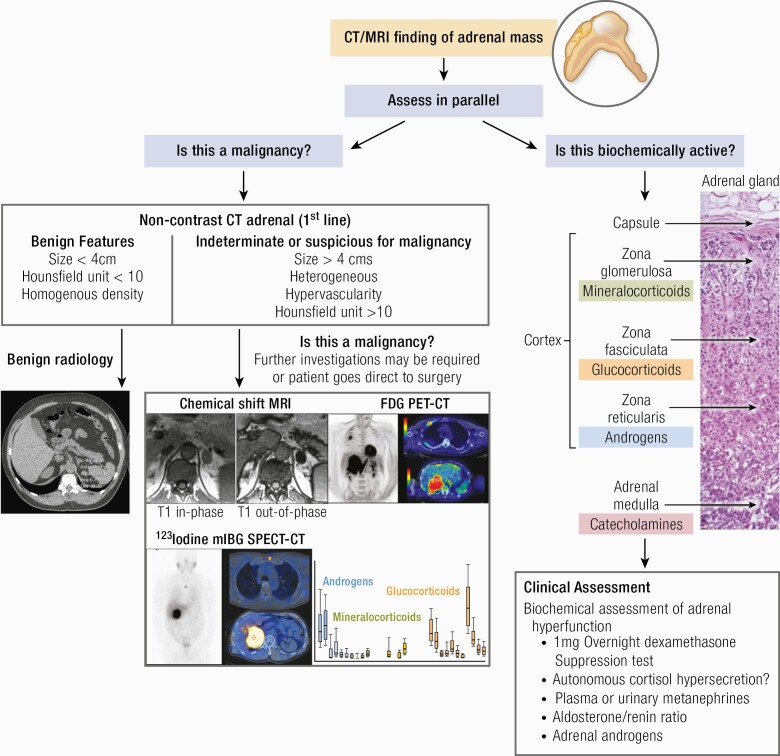

**Essential Points**

- Adrenal incidentaloma (AI) is a common endocrine diagnosis affecting ~2% of the general population, but over 7% of those over 70 years. It is rare in subjects below 40 years of age
- ~2% of patients with AI have adrenocortical cancer
- Up to 10% of patients with AI have autonomous secretion of adrenal hormones
- The initial investigation of choice is an unenhanced computed tomography scan of both adrenal glands
- Pheochromocytoma and autonomous cortisol secretion should be excluded in every case and aldosteronism in patients with underlying hypertension and/or hypokalemia
- Most patients with AI can be discharged once malignancy and hormone hypersecretion have been excluded
- A causative link between cortisol hypersecretion and age-related comorbidities should be firmly established before recommending surgical excision

The use of diagnostic imaging has increased dramatically over the last 3 decades, driven by several factors, including technological advancement in imaging modalities, growing awareness of preventive care, the rising number of diagnostic imaging centers, and increasing prevalence of chronic disease driven in large part by an aging population. According to estimates from the Organisation for Economic Co-operation and Development, in 2016, the United States performed 245 computed tomography (CT) scans per 1000 population compared with a mean of 151 per 1000 population across 11 other high-income countries (1). For magnetic resonance imaging (MRI) the figures were 118 per 1000 population in the US and 82 per 1000 in other countries (1). Therefore, approximately 80 million CT scans are performed each year in the US (2), and in the UK over 5 million CT scans were performed in 2018 (3). Improvements in imaging modalities and their increasing use have led to the increased discovery of unexpected pathological findings. One of the most common unexpected findings revealed by CT, MRI, or ultrasonography is an incidental adrenal mass or incidentaloma. An adrenal incidentaloma (AI) is defined as a clinically unapparent adrenal mass greater than 1 cm in diameter detected during imaging performed for reasons other than for suspected adrenal disease (4). The term “incidentaloma” was coined in 1982 by Geelhoed and Druy (5), who recognized that with the advent of improved resolution of radiological techniques clinicians were faced with the unfamiliar dilemma of early diagnosis of an asymptomatic adrenal mass. This strict definition, recognized by the European Society of Endocrinology and European Network for the Study of Adrenal Tumors (ESE/ENSAT), excludes adrenal lesions discovered during the screening of patients with hereditary syndromes or extra-adrenal tumors (4). Current guidelines do not recommend that incidentally discovered adrenal lesions with a diameter <1 cm undergo further investigation unless clinically indicated (4,6-8).

## Prevalence and epidemiology of AI

The prevalence of AIs varies depending on the source of data (autopsy, surgery, or radiology series) and patient selection (from general or specialized units). In the autopsy series with large patient numbers (series with greater than 1000 patients) (9-16), the reported prevalence of AI ranges from 1.05% (9) to 8.7% (10) (Table 1 (9-31)). In a large retrospective Japanese study, Kobayashi et al. investigated all cases of primary adrenocortical tumors recorded in the Pathological Autopsy Case Annuals of Japan during the 12-year period from 1973 to 1984 (17) (n = 321 847 cases) and reported a significantly lower overall prevalence of 0.03% of adrenocortical adenomas compared with historical series; however, the prevalence increased with age, with a peak in the fifth and sixth decades. Of the total number of identified adenomas in this autopsy series (n = 101), only 25 were identified in patients <50 years, while the remaining 75% were identified in patients older than 50 years. The variability in reported prevalence among the postmortem series reflects a combination of patient selection, inclusion criteria and the diagnostic challenge of distinguishing nodular hyperplasia and small adrenal nodules or adenomas. There is some overlap in the literature between the terms adenoma and nodule. Some authors have tried to define and divide these further. Russell et al. used the terms “adenoma” and “nodule” interchangeably to describe grossly visible collections of adrenal cortical cells, without any strict measurement criteria, in an autopsy series of 35 000 cases (15). They reported an overall prevalence of adrenal cortical adenomas (of all sizes) of 1.73%, increasing to 3.03% in patients older than 20 years (15). In a further autopsy series of 498 cases, Reinhard et al. (30) reported a higher prevalence of 5% for adrenal adenomas.

**Table 1.** Summary of world literature of prevalence of adrenal incidentaloma based on autopsy series.

Study/year (ref.)	Total no. of patients	Adenoma frequency (%)
Rinehart et al. 1941 (18)	100	3
Dempsey 1942 (19)	50	8
Russi et al. 1945 (11)	9000	1.45
Commons and Callaway 1948 (12)	7437	2.86
Schroeder 1953 (13)	4000	1.38
Dawson 1956 (20)	45	8.9
Holmes et al. 1956 (21)	53	1.9
Shamma et al. 1958 (22)	220	1.8
Spain and Weinsaft 1964 (23)	200	15.5
Dévényi 1967 (14)	5120	3.55
Kokko et al. 1967 (9)	2000	1.05
Hedeland et al. 1968 (10)	739	8.7
Dobbie 1969 (24)	50	32
Yamada and Fukunaga 1969 (25)	948	5.4
Granger and Genest 1970 (26)	2425	2.52
Russell et al 1972 (15)	35000	1.73
Abecassis et al. 1985 (27)	988	1.9
Winkelmann et al. 1987 (28)	117	3.4
Meagher et al. 1988 (29)	2951	5
Kawano et al. 1989 (16)	153000	0.24
Kobayashi et al. 1991 (17)	321847	0.03
Reinhard et al. 1996 (30)	498	5
Sington et al. 1999 (31)	29	6.9
**Median values for all studies**	948	3

Source data is highly variable in terms of autopsy, surgery or radiological in origin, patient selection and AI definition.

The prevalence of any disease increases with the observer’s ability to detect the abnormalities associated with the disease (32). The first CT scan series regarding AIs, published between 1982 and 1986, reported a prevalence of 0.6% to 1.3% (33-35), which likely represents an underestimation due to the low-resolution technology available at the time and failure to detect smaller lesions. Over the last 2 decades, there has been a dramatic increase in the detection of AIs; series with contemporary high-resolution CT techniques report a prevalence close to that observed at autopsy (Table 2 (36-44)). In 2006, Bovio et al. (41) reported a frequency of AIs of 4.4% in a prospective study of 520 patients. This was significantly higher than the previous imaging series. While this increase may reflect improved modern scanning technology, the study included only patients older than 55 years of age, thereby only capturing patients at an age at which there is a greater prevalence of adrenal masses. Similarly, Song et al. reported a prevalence of 5% for AI in a retrospective study of CT reports (42). However, the study protocol included a dedicated radiological review of adrenal imaging in a significant percentage of cases, thereby possibly resulting in the diagnosis of more AI than would have been encountered in routine radiological clinical practice (44,45).

**Table 2.** Prevalence of incidentally discovered adrenal masses by computed tomography (CT).

Study (ref)	Year	No. of CT scans	No. of adrenal masses	Frequency (%)
Glazer et al. (33)	1982	2200	14	0.6
Prinz et al. (34)	1982	1423	4	0.3
Abecassis et al. (27)	1985	1459	19	1.3
Belldegrun et al. (35)	1986	12000	88	0.7
Herrera et al. (39)	1991	61054	259	3.4
Caplan et al. (40)	1994	1779	33	1.9
Bovio et al. (41)	2006	520	23	4.4
Song et al. (42)	2008	65,231	3307	5.1
Hammarstedt et al. (43)	2010	34044	534	4.5
Davenport et al. (44)	2011	3705	37	1.0
Davenport et al. (36)	2014	4028	75	1.9
Maher et al. (37)	2018	38848	804	2.1
Taya et al. (38)	2019	42575	969	2.3
**Median values for all studies**	N/A	4028	75	1.9

Source data is highly variable in terms of autopsy, surgery or radiological in origin, patient selection and AI definition.

### Effect of age, sex, and ethnicity on AI prevalence.

Among autopsy and radiology series, the prevalence of AIs increases with age, showing a peak incidence in the fifth to seventh decades (Table 3 (39,40,42,46-84)). AIs are rarely seen in patients less than 30 years of age (66) and therefore if present should be investigated promptly due to the risk of adrenocortical carcinoma (ACC) or functional lesions. Previous imaging studies have reported that AIs are more common in females (66,71,73); however, this has not been observed in autopsy studies (11,85).

**Table 3.** Characteristics of adrenal incidentalomas: age, sex balance, tumor size and lateralization.

Study (ref)	Year	No. of patients	Mean Age (years)	Female/Male	Tumor size (median, mm)	Unilateral	Right	Left	Bilateral
Virkkala et al. (46)	1989	20	59	1.4	23	14	N/A	N/A	5
Herrera et al. (39)	1991	342	62	1.5	25	N/A			
Aso and Homma (47)	1992	210	53	0.7	49	N/A			
Reincke et al. (48)	1992	68	59	1.6	32	N/A			
Caplan et al. (40)	1994	26	66	1.9	NR	N/A			
Osella et al. (49)	1994	45	58	1.4	30	N/A			
Seppel and Schlaghecke (50)	1994	52	56	1.7	30	N/A			
Ambrosi et al. (51)	1995	32	55	2.5	27	27	22	5	5
Bencsik et al. (52)	1995	63	27—85	1.1	14—25	N/A			
Flecchia et al. (53)	1995	32	57	1.3	37	N/A			
Bardet et al. (54)	1996	35	N/A	N/A	26	35	N/A	N/A	0
Linos et al. (55)	1996	57	49	1.3	59	N/A			
Bastounis et al. (56)	1997	86	61	1.5	41	81	45	36	5
Bondanelli et al. (57)	1997	38	58	1.4	26	28	16	12	10
Kasperlik-Zeluska et al. (58)	1997	208	52	2.5	8—210	172	106	66	36
Terzolo et al. (59)	1995	45	55	1.4	31	N/A			
Proye et al. (60)	1998	103	N/A	N/A	N/A	N/A			
Murai et al. (61)	1999	59	N/A	N/A	N/A	N/A			
Tütüncü and Gedik (62)	1999	33	51	1.2	51	N/A			
Favia et al. (63)	2000	158	58	1.2	44	158	74	84	0
Mantero and Arnaldi (64)	2000	208	N/A	N/A	N/A	N/A			
Rossi et al. (65)	2000	50	54	1.7	33	47	28	19	3
Mantero et al. (66)	2000	1004	58	1.4	30	903	596	307	101
Tanabe et al. (67)	2001	38	N/A	N/A	N/A	N/A			
Barzon et al. (68)	2002	284	56	1.5	36	N/A			
Bülow and Ahrén (84)	2002	381	64	1.3	30	359	N/A	N/A	22
Tsvetov et al. (69)	2007	100	62	0.9	24	77	34	43	23
Bhargav et al. (70)	2008	53	41	1.1	78	51	30	21	2
Kasperlik-Załuska et al. (71)	2008	1444	10—87	2.7	10—230	1175	709	466	269
Song et al. (42)	2008	3307	64	N/A	20	N/A			
Vassilatou et al. (72)	2009	77	57	2.5	25	59	22	37	17
Comlekci et al. (73)	2010	376	55	2.3	25	313	N/A	N/A	54
Anagnostis et al. (74)	2010	64	62	1.9	N/A	N/A			
Giordano et al. (75)	2010	118	62	1.5	22	102	57	45	16
Yener et al. (76)	2010	317	55	2.7	24	362	190	172	45
Muth et al. (77)	2011	226	67	1	24.1	N/A			
Cho et al. (78)	2013	282	57	0.7	23	261	103	158	21
Di Dalmazi et al. (79)	2014	129	61	1.5	21 (max)	92	N/A	N/A	22
Hong et al. (80)	2017	1149	54	0.8	18	N/A			
Ahn et al. (81)	2018	1005	44	0.7	17	902	308	594	103
Akkuş et al. (82)	2018	229	52	1.7	48	202	N/A	N/A	27
Goh et al. (83)	2018	228	61	1.5	18	196	70	126	32
**Median value for all studies**		64	57.5	—	30	—	**—**	**—**	**—**

Abbreviation: N/A, data not available.

A recent large prospective Korean study, the COAR (the Co-work Of Adrenal Research) (81), investigated the characteristics of 1005 Korean patients with AIs and compared them with those of the largest previous retrospective study conducted in a Study Group on Adrenal Tumors of the Italian Society of Endocrinology cohort (66). The results showed some discordance between the patient cohorts. AIs in Korean patients were more frequent in men (57%) and the population was younger (median 55 years) than the Italian patient cohort. Also, fewer AIs underwent surgical resection in the Korean study. These differences likely reflect the different time period of the studies: the patients of the Italian study were recruited from 1980 to 1995, while those of the COAR were recruited from 2011 to 2014. The fact that fewer patients in the COAR study underwent adrenalectomy may be due to smaller adenoma size at discovery, an increase in the detailed characterization of AIs using improved imaging techniques (discussed in “Imaging Evaluation of an AI”), and endocrinological assessment (discussed in “Endocrine Work-up of AI”) leading to more reassurance regarding their benign nature. There has also been an increase in our knowledge concerning the natural history of untreated adrenal lesions and the recent development of international evidence-based management guidelines reflect this (4,6).

### Size and lateralization of AI.

A large number of clinical studies have investigated the characteristics of AIs (Table 3 (39, 40,42,46-84)). The mean diameter of AI discovered by CT scan is 30 mm, ranging from 8 to 230 mm (Table 3 (39,40,42,46-84)) (the 8-mm lesions reflect the results of older studies before adopting 1 cm as the threshold for the definition of an AI). Many studies are limited by their retrospective nature, specialized center bias, patient selection bias, recall bias, and small sample size. However, it should be noted that all studies consistently report a higher incidence of ACC if the adrenal mass is greater than 4 cm in size (39,49,63,66,71).

The development of new CT protocols has improved the detection and characterization of adrenal masses (87). A similar distribution of lesions in the right and left adrenal gland has been reported in many CT series, in keeping with autopsy studies (Table 3). However, some studies suggest a higher prevalence of left-sided adrenal tumors detected on imaging (81,88,89), for example, the COAR study cohort described above (81). This observation may reflect a detection bias as left-sided adrenal tumors may be more readily apparent to the radiologist. Similarly, a recent large cross-sectional study of abdominal CT and MRI imaging in 1376 patients (90) reported a higher detection of left-sided adenomas than right-sided adenomas in each size category except when the tumor size was ≥30 mm. The authors concluded that this detection bias may result in under-recognition of small (<30 mm) right-sided lesions and bilateral disease (90).

## Anatomy and Physiology of the Adrenal Gland

### Anatomy of the adrenal gland

A brief overview of adrenal embryology, anatomy, physiology, and pathology is required to appreciate the clinical significance of AIs as it relates to the investigation and treatment of these lesions.

### Historical perspective

The anatomy of the adrenal gland was first described by Bartholomeo Eustacius in 1563 and its functional importance subsequently elucidated by the pioneering work of Thomas Addison in 1855 (91). Shortly after Addison’s work, Brown-Séquard performed a series of bilateral adrenalectomies in several species of small animals and demonstrated that the adrenal glands are essential for life (92). Improvement in microscopy techniques in the nineteenth century helped describe the anatomy of the adrenals (Kölliker 1852), and the distinction between “cortical and medullary substances” (93).

### Embryology and development

**Figure 1.**
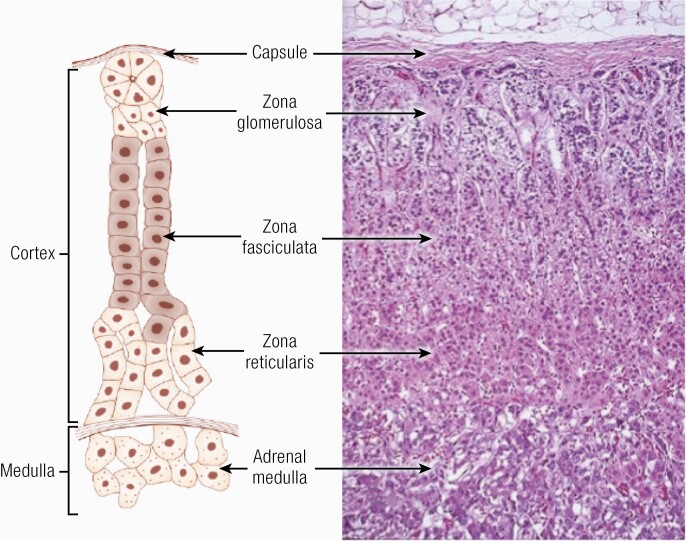
Schematic diagram of the structure of the human adrenal cortex (92). Adapted from PM Stewart, Chapter 14 The Adrenal Cortex. In Williams Textbook of Endocrinology, 10th ed. , Copyright © 2003 Elsevier Reproduced from Williams RH, Larsen PR. Williams Textbook of Endocrinology. 10th ed./ P. Reed Larsen….[et al.] ed. United States: Philadelphia : Saunders, ©2003. Copyright © 2003 Elsevier.

The adrenal gland is derived from 2 embryologically distinct origins with the adrenal cortex arising from the coelomic mesoderm of the urogenital ridge and the medulla from the neuroectoderm (neural crest cells) (92). The fetal adrenal gland is evident from 6 to 8 weeks of gestation and rapidly increases in size so that by midgestation it is larger than its adjacent kidney. In later stages of embryonic development, the cortex engulfs and eventually encapsulates the entire medulla. The adrenal cortex differentiates fully into its 3 constitutive zones (zona glomerulosa, zona fasciculata, and zona reticularis) by 3 years of age (94, 95) (Fig. 1).

The development of the adrenal cortex is dependent on the blood supply, paracrine adrenal factors, hormonal factors, and adrenocortical innervation (96). The nuclear receptor steroidogenic factor-1 (SF1 also known as NR5A1) is a pivotal factor for the initiation and fetal maturation of the adrenal cortex (97), with its absence resulting in adrenal aplasia (98). An interplay between the transcription factors SF1 and an SF1 target gene, *DAX 1*, determines the extent to which steroidogenic enzymes are induced, and adrenocortical cells become differentiated (97). While adrenocortical growth and differentiation are independent of adrenocorticotropin (ACTH) during the first trimester of pregnancy, ACTH begins to play an essential role in the morphological and functional development of the adrenal gland after 15 weeks of gestation.

### Structure, vasculature, and innervation of the adrenal gland

**Figure 2.**
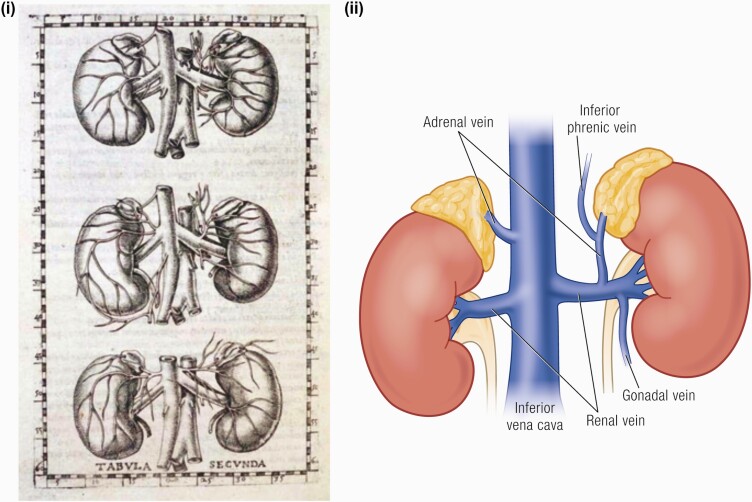
Anatomy of the adrenal gland. (i) Eustachio’s original drawing from Tabulae anatomicae published by Johannes Maria Lancisi in 1714 (99). (ii) The left adrenal–renal venous complex. The right adrenal vein (AV) drains directly into the IVC (100).

The adult adrenal gland is a pyramidal structure weighing approximately 4 grams, and is approximately 2 cm wide, 5 cm long, and 1 cm thick. It lies immediately above the kidney on its posteromedial surface (96). Although the left and right adrenal glands are symmetrical, the left gland is in a more caudal position, lying anteromedially to the left renal upper pole, laterally to the aorta and left diaphragmatic crus, and superior to the left renal vein (Fig. 2).

Beneath the adrenal capsule, the zona glomerulosa, constitutes approximately 15% of the mass of the cortex (depending upon salt intake), (Fig. 1 (92)). The zona fasciculata constitutes 75% of the cortex comprising lipid-laden cells that are larger than those in the zona glomerulosa, organized into bundles, leading to the origin of its name “fascicles.” The innermost layer of the adrenal cortex is the zona reticularis comprising irregular cells arranged as cords with little lipid content (92).

Though small, the adrenal glands have an extensive vasculature. This may account for the predilection of cancer metastases to the adrenal gland (101) and also for its susceptibility to nontraumatic adrenal hemorrhage (102). Three arteries supply each adrenal gland: the superior suprarenal artery from the inferior phrenic artery, the middle suprarenal artery directly from the abdominal aorta, and the inferior suprarenal artery from the renal artery. Blood is channeled into the subcapsular arteriolar plexus and subsequently distributed to the sinusoids, which in turn supply the adrenal cortex and medulla (96, 103) (Fig. 2). There are numerous variations of the venous drainage of the adrenal gland based on the adrenal and renal vein configurations and combinations, which have been extensively reviewed by Cesmebasi et al. (100). These variations are important both for the surgical management of adrenal lesions but also for diagnostic adrenal vein sampling (100).

### Physiology of the adrenal gland

#### Functional zonation of the adrenal cortex.

Aldosterone is the primary mineralocorticoid produced by the zona glomerulosa cells under the control of angiotensin II and extracellular potassium. The daily production rate of aldosterone varies between 80 and 200 μg/day (104), depending on daily salt intake. Aldosterone functions as the ligand for the mineralocorticoid receptor in target tissues that include the colon, salivary gland, and the renal distal convoluted tubule and collecting ducts, where it causes increased reabsorption of sodium and increased excretion of both potassium (by principal cells) and hydrogen ions (by intercalated cells of the collecting duct). Aldosterone secretion is confined to the outer zona glomerulosa due to the restricted expression of *CYP11B2*.

Glucocorticoids are secreted in relatively high amounts (cortisol 10-20 mg/day) from the zona fasciculata cells under the control of ACTH secreted from the anterior pituitary (92). In humans, cortisol is the main glucocorticoid produced by the adrenal cortex under normal conditions and its actions include mobilization of fats, proteins, and carbohydrates. Once produced and released into the bloodstream, glucocorticoids facilitate the release of energy stores for utilization during stress. Integral to the feedback control of the activated hypothalamic–pituitary–adrenal (HPA) axis, glucocorticoids inhibit the production and secretion of both corticotropin-releasing hormone (CRH) and ACTH from the HPA axis. As a class, adrenal androgens (dehydroepiandrosterone [DHEA], DHEA sulfate [DHEAS], and androstenedione) are the most abundant steroids secreted from the adult adrenal gland (>20 mg/day). DHEA is sulfated only in the zona reticularis to form DHEAS.

#### Steroidogenesis.

**Figure 3.**
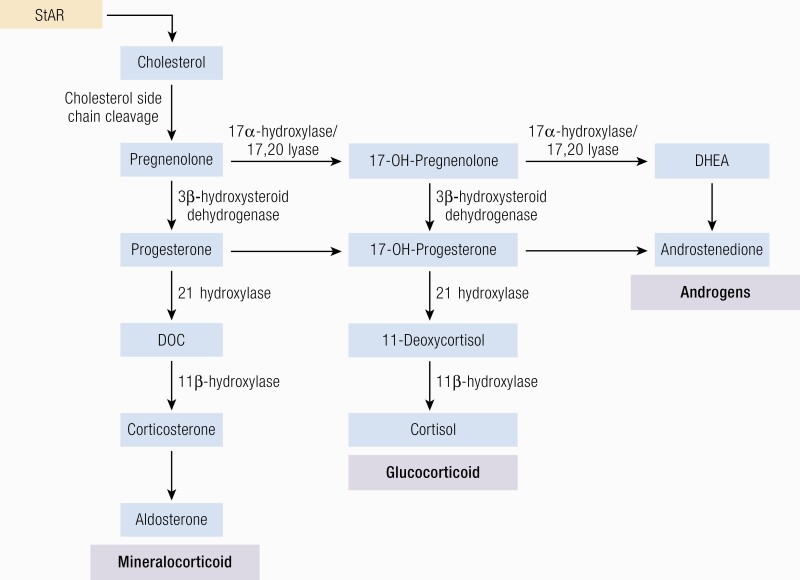
Adrenal steroidogenesis. After the steroidogenic acute regulatory (StAR) protein-mediated uptake of cholesterol into mitochondria within adrenocortical cells, aldosterone, cortisol, and adrenal androgens are synthesized through the coordinated action of a series of steroidogenic enzymes in a zone-specific fashion. A'dione, androstenedione; DHEA, dehydroepiandrosterone; DOC, deoxycorticosterone. PM Stewart, Chapter 14 The Adrenal Cortex. In Williams Textbook of Endocrinology, 10th ed. , Copyright © 2003 Elsevier Reproduced from van Berkel A, Lenders JW, Timmers HJ. Diagnosis of endocrine disease: Biochemical diagnosis of phaeochromocytoma and paraganglioma. Eur J Endocrinol 2014; 170:R109-119.

Three main subclasses of steroid hormones are produced by the adrenal cortex: glucocorticoids (cortisol, corticosterone), mineralocorticoids (aldosterone, deoxycorticosterone [DOC]), and adrenal androgen and their precursors [mainly DHEA, DHEAS, and androstenedione]. Steroid hormones regulate a wide variety of developmental and physiological processes from fetal life to adulthood. Steroid hormones are all synthesized from cholesterol and hence have closely related structures based on the classic cyclopentanophenanthrene 4-ring structure. The physiology of human steroidogenesis has been extensively described previously by Miller et al. (105) and the biochemical pathways involved in adrenal steroidogenesis are shown in Fig. 3, to highlight their increasing importance in clinical practice as a diagnostic tool (discussed in “The Future: Investigation and Management”). As depicted, steroidogenesis involves the collaborative action of a series of enzymes including cytochrome P450 enzymes following the transport of intracellular cholesterol into the adrenal cortex under stimulation from ACTH (92,106).

Importantly, the functional zonation of the adrenal cortex is dependent upon the zonal expression of 2 key enzymes: the final step in cortisol biosynthesis involves the conversion of 11-deoxycortisol to cortisol by the enzyme 11β-hydroxylase (*CYP11B1*), largely in the zona fasciculata. By contrast in the zona glomerulosa (although 11 β -hydroxylase may also convert deoxycorticosterone to corticosterone) it is the enzyme *CYP11B2* or aldosterone synthase that is uniquely required for the conversion of corticosterone to aldosterone through the intermediate 18-OH corticosterone.

#### Physiology of the adrenal medulla.

**Figure 4.**
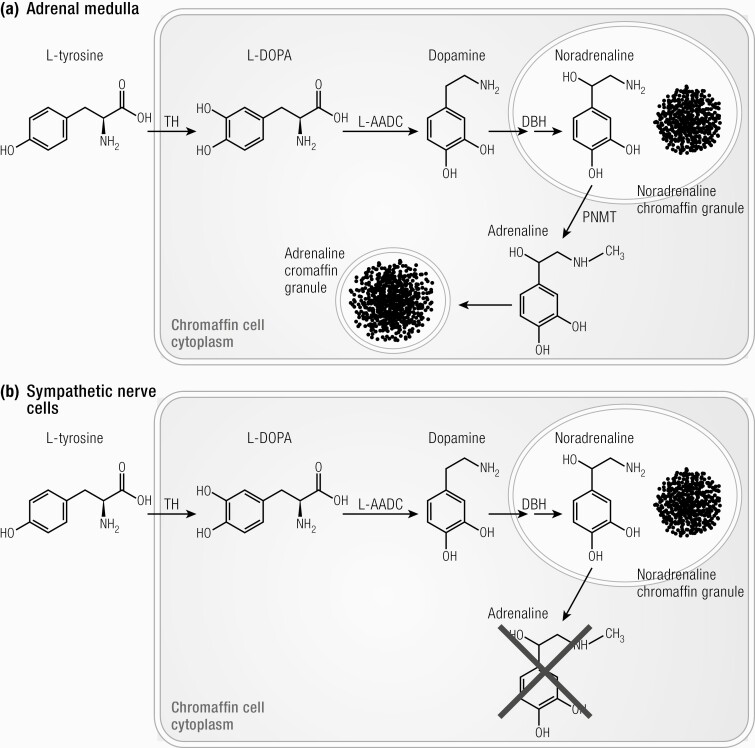
Biosynthesis of catecholamines in (A) chromaffin cells of adrenal medulla and (B) sympathetic nerve cells. TH, tyrosine hydroxylase; L-AADC, aromatic L-amino-acid decarboxylase; DBH, dopamine- β -hydroxylase; PNMT, phenylethanolamine-N-methyltransferase (108). Reproduced from van Berkel A, Lenders JW, Timmers HJ. Diagnosis of endocrine disease: Biochemical diagnosis of phaeochromocytoma and paraganglioma. Eur J Endocrinol 2014; 170:R109-119.

Understanding the biosynthesis pathways, metabolism, and breakdown of catecholamines is crucial to understanding the biochemical assessment of pheochromocytomas and paragangliomas (107, 108). Briefly, the biosynthesis of catecholamines starts with the conversion of amino acid L-tyrosine to L-3,4-dihydroxyphenylalanine (L-DOPA) by the enzyme tyrosine hydroxylase (TH). L-DOPA is converted to dopamine which is translocated to catecholamine storage vesicles of chromaffin cells of the adrenal medulla, sympathetic nerves, and paraganglia (Fig. 4 (108)). The enzyme dopamine- β hydroxylase is responsible for the conversion of dopamine into norepinephrine (noradrenaline). In adrenal medullary chromaffin cells, norepinephrine (noradrenaline) is further converted to epinephrine (adrenaline) by phenylethanolamine N-methyltransferase (Fig. 4 (108)). As this enzyme is only present in these cells, epinephrine (adrenaline) is almost exclusively produced within the adrenal medulla (108).

**Figure 5.**
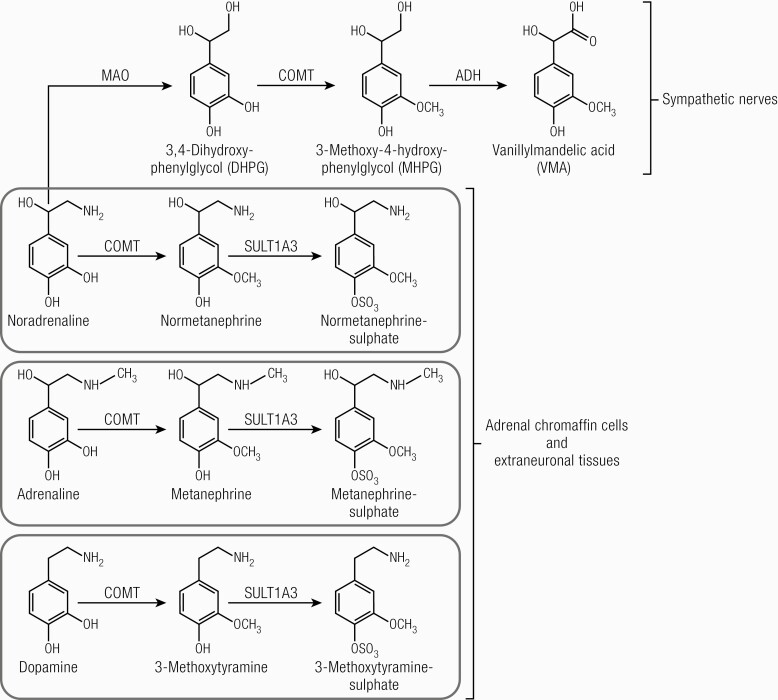
Metabolism of catecholamines. ADH, alcohol dehydrogenase; MAO, monoamine oxidase; COMT, catechol-O-methyltransferase; SULT1A3, sulfotransferase 1 A3. Adrenaline, epinephrine; noradrenaline, norepinephrine. (108). Reproduced from van Berkel A, Lenders JW, Timmers HJ. Diagnosis of endocrine disease: Biochemical diagnosis of phaeochromocytoma and paraganglioma. Eur J Endocrinol 2014; 170:R109-119.

Catecholamines are metabolized through several pathways, resulting in numerous metabolites (Fig. 5)). The majority of circulating norepinephrine (noradrenaline) is derived from noradrenergic neurons of the central and sympathetic nervous system (108). Deamination of neuronal norepinephrine (noradrenaline) to 3,4-dihydroxyphenylglycol occurs by monoamine oxidase. Norepinephrine (noradrenaline) is also partially metabolized in extraneuronal tissues and adrenal chromaffin cells, where it is converted to normetanephrine by catechol-O-methyltransferase (COMT) (108). Epinephrine (adrenaline) is mainly metabolized within adrenal chromaffin cells by COMT, resulting in the O-methylated metabolite metanephrine. Metabolism of dopamine follows other pathways, resulting in the production of the O-methylated metabolite methoxytyramine. Plasma free metanephrines are conjugated to sulfates by gut wall enzymes (108).

In patients with pheochromocytomas/paragangliomas, more than 90% of catecholamine catabolism occurs continuously within the tumor itself, mostly through the action of COMT (107). Elevated circulating levels of O-methylated metabolites, therefore, point to the presence of pheochromocytoma or paraganglioma with greater sensitivity and specificity than elevated levels of the parent amines. Elevation in amines may result from increased sympathetic nervous system activity or originate from other sources (107).

### Histology of adrenal tumors

This has been extensively reviewed by Erickson et al. (109). In brief, there are several methods for immunophenotyping adrenal tissue and tumors. Both adrenocortical tumors and pheochromocytomas stain positive for synaptophysin but adrenocortical tumors are negative for chromogranin (109). S100 protein usually highlights sustentacular cells in pheochromocytomas and is negative in adrenocortical tumors. Adrenocortical tumors are also usually (but not always) positive (and pheochromocytomas negative) for Cytokeratin CAM 5.2, α-inhibin and calretinin. Steroidogenic factor 1 is increasingly recognized as a key marker of adrenocortical cell lesions (109).

Both adrenocortical cancers and melanomas stain positive for Melan A, but adrenocortical tumors are negative for S100 (a positive marker for melanoma). Additional markers may be required to exclude other primary lesions, for example, thyroid transcription factor 1 and cytokeratin.

#### Differentiation of adrenocortical adenoma and carcinoma.

Although there are several clinical, endocrinological and radiological features that help risk stratify an adrenal lesion with regard to the likelihood of ACC (see “Etiology and clinical presentation of AI”), the final diagnosis is a histological one and requires the input of an experienced adrenal pathologist (110). The most widely used histological scoring system is the Weiss score (Table 4) based on 9 histological parameters (scored as 0 if absent and 1 if present) (110,111).

**Table 4.** Summary of Weiss score criteria.

Criteria	Scoring points	
	0	1
Nuclear grade (Fuhrmann nuclear grade system)	I/II	III/IV
Mitoses	<6/10 HPF	≥6/10 HPF
Atypical mitoses	−	+
Clear cell component	<25%	≥25%
Diffuse architecture	<1/3	≥1/3
Confluent necrosis	−	+
Venous invasion	−	+
Sinusoidal invasion	−	+
Capsular infiltration	−	+

Adapted from references (111-113).

Abbreviation: HPF, high power fields.

A threshold above a Weiss score of 3 (also on the modified score) was associated with an increased risk of malignant behavior (112). In the initial study by Weiss, the features with the strongest association to outcome were the mitotic rate >5/50 high powered field, atypical mitoses, and venous invasion (111) and in the second study by Weiss in 1989 the strongest association with patient outcome was the mitotic rate (112).

Another key predictor of malignant behavior is the Ki67 proliferative index (114). The cutoff at which a Ki67 labeling index confirms the diagnosis of ACC and predicts aggressive disease is debated and ranges from >2.5% to >7% (113,115-117). However, because of the enormous intratumoral heterogeneity of ACC, the site in which the Ki67 labeling index is obtained markedly influences the results. In particular, the question of whether the Ki67 labeling index should be calculated as the average of the entire tumor specimen or the summation of hot spots in the specimens has not been resolved (113). This limitation should be kept in mind when applying the Ki67 labeling index to categorizing and adrenal lesion and as a prognostic marker for ACC (113). The Helsinki score used in some centers incorporates the Ki67 labeling index and has been proposed to be a better predictor of outcome (118).

#### Pheochromocytoma of the Adrenal Gland Scaled Score and Grading of Adrenal Pheochromocytoma and Paraganglioma tool.

In 2002 Thompson described a tool, Pheochromocytoma of the Adrenal gland Scaled Score (PASS), based on the presence or absence of 12 specific histological features to allow better distinction between benign and malignant tumors (119). The histological features include vascular/capsular/periadrenal adipose tissue invasion, large nests or diffuse growth, focal or confluent necrosis, high cellularity, tumor cell spindling, cellular monotony, increased mitotic figures (>3/10 high power fields), atypical mitotic figures, nuclear pleomorphism, and hyperchromasia with weighted scores between 1 and 2. Tumors with a PASS score >4 were defined as having malignant potential, whereas those with a lower score were considered benign. Subsequent validation by a panel of experienced pathologists concluded that due to significant inter- and intraobserver variation PASS was not recommended for clinical prognostication (120).

Subsequently, Kimura et al. (121) developed a less involved scoring system for prediction of metastases, Grading of Adrenal Pheochromocytoma and Paraganglioma (GAPP) consisting of a smaller number of pathological parameters with weighted scoring (1 or 2 points) including histological pattern; cellularity; comedo necrosis; capsular/vascular invasion; Ki67 proliferative index; and catecholamine phenotype. A GAPP score of 0 to 2 is considered low risk, 3 to 6 intermediate risk, and 7 to 10 high risk. GAPP scoring was independently validated by Koh et al. and is considered a useful risk stratification tool for the prediction of metastatic potential (122).

## Etiology and Clinical Presentation of AI

Many AIs, while picked up incidentally, may have clinical symptoms or associated signs on closer questioning and clinical examination. The etiology of an AI is variable and includes tumors from the adrenal cortex, the adrenal medulla, and metastatic deposits.

### Etiology: tumors of the adrenal cortex

#### Adrenocortical adenoma.

An ACA is a benign neoplasm of adrenocortical cells. The majority of these lesions are nonsecretory; however, some may produce glucocorticoids independent of ACTH and mineralocorticoids independent of stimulation from the renin–angiotensin system. Rarely, they may also produce androgens or estrogens which may result in virilization or feminization. The 3′,5′-cyclic adenosine 5′-monohosphate–protein kinase A (cAMP–PKA) pathway is important for the regulation of adrenocortical cell development. ACTH binds to the ACTH receptor (a G-protein coupled receptor encoded for by the *MCR2* gene) in the adrenocortical cell, thereby activating adenylyl cyclase, cAMP synthesis and activation of PKA. Abnormally increased cAMP–PKA signaling is thought to be the key mechanism in the development of most benign adrenocortical tumors (123).

#### Cortisol-producing adenoma.

Autonomous cortisol secretion (ACS) (of variable clinical significance and severity) is frequently found in patients with an ACA. The majority of these cases are due to non-ACTH-dependent ACS from the adenoma. However, aberrant expression and activation of G-protein coupled receptors have been implicated as a possible mechanism explaining cortisol hypersecretion (and in primary hyperaldosteronism) (124, 125).

#### ACC, adrenal lymphoma, and adrenal metastases.

##### Adrenocortical carcinoma.

Primary ACC is rare, with an estimated population incidence of 1 to 2 per million per year (126,127). Between 40% and 60% of ACCs are functional and may present with symptoms of hormonal hypersecretion (126,127,128,129), most commonly with Cushing’s syndrome (estimated 45%). Approximately 25% of ACCs will cosecrete glucocorticoids and androgens. Solely androgen-secreting ACCs are less common (approximately 10%), usually presenting with virilization without features of glucocorticoid excess. Feminization and hyperaldosteronism are rare occurring in <10% of cases (127,130). Importantly, if there is evidence of hypersecretion from 2 adrenal zones this is highly suggestive of an ACC as benign lesions do not secrete in this pattern.

Approximately 30% of ACCs present with symptoms of local mass effect, such as abdominal or flank pain. As ACCs are retroperitoneal they may present late and have often reached a large size (131).

The impact of the functional status and clinical features of ACCs on survival is unclear (132,133). However, cortisol hypersecretion leading to clinically evident Cushing’s syndrome is recognized as a significant cause of morbidity and mortality (134,135) due to increased risk of infection, metabolic, bone, and vascular complications (136).

Malignancy is also suspected based on imaging characteristics of the adrenal mass and size. The size of the adrenal mass is predictive (but not 100%) of malignancy. In an Italian study of 887 patients with AIs, 90% of ACCs had a diameter of greater than 4 cm at presentation, with a 4-cm cut-off having a 93% sensitivity for detecting ACC (64). Size at presentation may also impact prognosis from ACC, with a smaller size of tumor at diagnosis associated with a significantly higher 5-year survival (137). Imaging characteristics of adrenal lesions will be discussed in detail in “Imaging evaluation of an AI.”

Mutations in β -catenin (*CTNNB1*) leading to constitutive activation of the Wnt signaling pathway is a frequent finding in benign and malignant adrenocortical tumors (138). The Wnt signaling pathway is thought to be important in the embryonic development of the adrenal gland (139). Constitutive activation of this pathway is also implicated in cancer development in several other organs (140). In 1 study of 100 adrenal adenomas that had been surgically excised, 36% were found to contain *CTNNB1* mutations (141). Somatic activating mutations of *GNAS*, which encodes the α-subunit of the stimulatory G protein (G_S_α), occur in 5% to 17% of adrenal adenomas which are cortisol secreting (142,143). An example of this is constitutive activation of adenylyl cyclase as a result of somatic *GNAS* mutations in McCune–Albright syndrome (144).

Inactivating mutations in *PRKAR1A* have been described in cortisol-producing adrenal tumors. *PRKAR1A* gene encodes for a regulatory subunit of PKA, and inactivating mutations lead to constitutive activation of the cAMP–PKA pathway. Although mutations in this gene were first described in Carney complex (145), somatic mutations of *PRKAR1A* have been described in some sporadic adrenocortical tumors (146).

Somatic activating mutations of protein kinase A catalytic subunit (*PRKACA*) has been implicated in up to 50% of patients with adenomas with clinical Cushing’s syndrome, but not in adenomas producing less cortisol (138,142,147). Mutations in *PRKACA* may be associated with smaller adenomas but higher levels of cortisol production than adenomas where this mutation is not present (143,148). The lower frequency of these mutations in adenomas which produce less cortisol may be an explanation for the lack of progression in these patients to a clinically apparent Cushing’s syndrome.

Mutations in cyclic nucleotide phosphodiesterase have also been noted in cortisol-producing ACAs. These are enzymes that breakdown cyclic nucleotides and as a consequence regulate cAMP levels and cAMP–PKA pathway activity. Mutations in *PDE11A* and *PDE8B* genes are the most commonly reported (149,150).

#### Aldosterone-producing adenoma.

Mutations in *KCNJ5* (potassium channel) have been documented in patients with aldosterone-producing adenomas in approximately 40% of patients (151) from European cohorts, though much higher rates are reported in patients from Japan and Asia (152,153). These mutations lead to increased sodium conductance and cellular depolarization leading to calcium influx, increased intracellular calcium signaling, and increased *CYP11B2* mRNA expression with increased aldosterone production and glomerulosa cell proliferation (123). Adenomas with *KCNJ5* mutations tend to be larger than those which do not carry the mutation and appear to be more common in women than men (154).

Other somatic mutations in several of the genes involved in the regulation of aldosterone production have also been identified (155). In aldosterone-producing adenomas that did not have *KCNJ5* mutations, abnormalities in *ATP1A1* (encoding a Na^+^/K^+^ ATPase α subunit) were found in 5.2% and of *ATP2B3* (encoding a Ca2^+^ ATPase) in 1.6%, with these mutations associated with increased plasma aldosterone concentrations and lower potassium concentrations than cases without the mutation (156). Additionally, mutations in the *CACNA1D* gene have been identified and recently gain of function mutations in the *CLCN2* chloride channel gene (157) has also been described. This gene encodes a voltage-gated calcium channel and 11% of aldosterone-producing adenomas without mutations in *KCNJ5* have been reported to carry mutations in this gene (158). Mutations in *KCNJ5*, *CACNA1H*, *ATP1A1*, *ATP2B3*, and *CACNA1D* account for approximately 50% of aldosterone-producing adrenal adenomas (159) in patients from Europe; this is likely to be higher in Asian patients.

#### Adrenocortical carcinoma.

ACC is rare and aggressive and can occur at any age. However, peak incidence tends to be before the age of 5 and between the ages of 40 and 60 years of age (126,127). Most cases of ACC are sporadic but some are known to occur with other tumor syndromes (160). The most well known of these are Li–Fraumeni syndrome (*TP53* gene), Multiple Endocrine Neoplasia type I (*MEN1* gene) and Beckwith–Weidemann syndrome (abnormalities in *11p15I* gene). The genetic mutations in these syndromes are well characterized. ACCs have also been noted in familial adenomatous polyposis (*FAP* gene), neurofibromatosis type 1 (*NF1* gene), and Carney complex (*PRKAR1A* gene) (161).

In contrast, the genetic basis for sporadic ACCs is less clear. Loss of heterozygosity of chromosome 17p13, which codes for the tumor suppressor gene *TP53*, is a common finding in sporadic ACCs (162). However, only one-third of these ACCs have a mutation of *TP53* (163). As previously described, mutations in *CTNNB1* are also present in ACCs and may be associated with poor outcome (164). *ZNRF3* is thought to be a tumor suppressor gene related to the β -catenin pathway, encoding a cell surface E3 ubiquitin ligase. Mutations in this gene were identified in 21% of 123 ACCs following genomic characterization (165).

Loss of heterozygosity at the 11p15 locus can lead to insulin-like growth factor-2 overexpression which is associated with malignant ACCs (166,167). Pan-genomic characterization of ACCs has identified several other driver genes including *CDKN2A*, *RB1*, *DAXX*, *TERT*, *MED12*, *PRKAR1A*, *RPL22*, *TERF2*, *CCNE1*, and *NF-1* (165,168).

#### Bilateral adrenocortical tumors.

Although the majority of AIs are unilateral, bilateral AIs may be found in 10% to 15% of cases (169). Two large studies (169,170) found that the most common causes of bilateral AI were metastasis, primary bilateral macronodular adrenal hyperplasia (PBMAH), and bilateral cortical adenomas. Other causes of bilateral AI include bilateral pheochromocytomas, congenital adrenal hyperplasia (CAH), Cushing’s disease, or ectopic ACTH secretion with secondary bilateral adrenal hyperplasia.

Bilateral adrenal hyperplasia is characterized by a nodule diameter of less than 1 cm (micronodular) and greater than 1 cm (macronodular). Primary pigmented nodular adrenocortical disease is characterized by multiple pigmented micronodules and does not usually present as an AI, and therefore will not be discussed further in this review.

PBMAH presents on imaging with characteristic multiple bilateral macronodules. Whole-genome sequencing, along with single nucleotide polymorphism array analyses, identified recurrent mutations in an armadillo repeat containing 5 (*ARMC5)* gene, located in chromosome 16p, in 50% of BMAH patients who underwent surgery (171). Patients with *ARMC5* mutations tend to display adrenal hyperplasia associated with multiple nodules (172-175). Discovering *ARMC5* mutations was the first evidence of BMAH as a genetic disease. *ARMC5* is a putative tumor suppressor, with 2 hits in each adrenal nodule: a first germline alteration, found in leukocyte DNA and common to all nodules, and a second somatic hit (171). The penetrance of *ARMC5* mutations is variable (172). *ARMC5* overexpression induces apoptosis in vitro and its inactivation decreases steroidogenesis (171). The pathophysiology of BMAH is discussed further in “Bilateral AI and ACS.”

### Etiology: tumors of the adrenal medulla

Tumors that originate from the chromaffin cells of the adrenal medulla, which secrete catecholamines, are termed pheochromocytomas. They may be benign or malignant. They can occur at any age, although are most common in the fourth to the fifth decade (176). The annual incidence of pheochromocytoma is estimated at 0.8 per 100 000 person-years (177). It is estimated 40% of catecholamine-secreting tumors are part of a hereditary syndrome and many endocrinologists are now offering genetic analysis to all affected cases. Von Hippel Lindau (VHL), multiple endocrine neoplasia (MEN) type 2, and NF1 are the most well known of these and all have an autosomal dominant inheritance.

Other mutations have been associated with sporadic pheochromocytoma clustering to 2 common pathways: hypoxic signaling and kinase signaling genes. Affected genes in cluster 1 (hypoxic pathway) include subunits of succinate dehydrogenase (*SDHD*, *SDHC*, *SDHB*, *SDHA*, *SDHAF2*), *HIF-1alpha, EGLN1/2*, and *KIF1B* in addition to *VHL.* Cluster 2 genes code for activating proteins in kinase signaling and include *NF1*, *RET*, *MAX*, and *TNEM127*. Cluster 2 gene mutations are more likely to result in adrenal pheochromocytomas, whereas those in Cluster 1 mostly result in extra-adrenal noradrenergic paragangliomas (except for mutations in *VHL*). Mutations in *VHL*, *RET*, *NF1*, *SDHB*, and *SDHD* account for 90% of all pheochromocytomas and paragangliomas (123).

Hereditary pheochromocytomas tend to be diagnosed at a younger age, because of routine biochemical screening or genetic testing and are also more likely to be bilateral. In contrast, sporadic pheochromocytomas may be discovered incidentally on adrenal imaging (178,179). *SDHB* (10.3%) and *SDHD* (8.9%) mutations are the most frequent germline mutations in pheochromocytoma and paragangliomas (180).

The relative production of plasma metanephrine compared with plasma normetanephrine and methoxytyramine may help distinguish the genetic etiology of pheochromocytoma. In 1 large study of patients with pheochromocytoma and/or paraganglioma, patients with *NF1* and *MEN2* mutations could be discriminated from those with *VHL* and *SDH* mutations in 99% of cases by the relative concentrations of normetanephrine and metanephrine, as all patients with NF1 and MEN2 presented with tumors characterized by increased plasma concentrations of metanephrine, in contrast to patients with *VHL* and *SDH* mutations, usually presenting with increases in normetanephrine or methoxytyramine. Additionally, measurements of plasma methoxytyramine discriminated patients with *SDH* mutations from those with *VHL* mutations in a further 78% of cases (181).

### Clinical presentation of AI

By definition, an AI is discovered when imaging is performed for another indication without any obvious clinical features of adrenal disease. However, on closer history and examination following the discovery of the adrenal lesion, there may be features suggestive of adrenal disease. In cases where an adrenal mass is large (causing local pressure effect) or the tumor demonstrates clinically significant hormone hypersecretion, specific clinical features may be uncovered. However, it is important to note that even functional tumors may not result in significant clinical symptoms. Imaging characteristics cannot reliably distinguish between functional and nonfunctional tumors. As such, all patients with an AI should be systematically assessed for functional activity by endocrine biochemical testing, regardless of the presence or absence of symptoms (4).

#### Adrenal lymphoma.

Adrenal gland involvement is well recognized and seen in up to 25% of patients with non-Hodgkin lymphoma usually as part of disseminated disease (182). Conversely primary adrenal lymphoma is much rarer accounting for <1% of all cases of non-Hodgkin lymphoma (183). Primary adrenal lymphoma usually manifests as a large well-defined mass with homogeneous enhancement and may invade into surrounding structures and biopsy may be required to differentiate from other more common tumors (184).

#### Adrenal metastases.

A metastatic tumor is a common finding in the adrenal gland at postmortem and the predilection of the adrenal gland for metastatic deposits is thought to be due to its extensive sinusoidal blood supply (185). In one series of 464 patients with metastatic adrenal gland lesions, 90% were carcinomas, with the others being hematological, sarcomas, or melanomas. Of the carcinomas, the most common primary site in 35% of cases was lung, followed by gastrointestinal, kidney, and breast. Interestingly, bilateral adrenal involvement was noted in almost half the patients in this case series (186). Adrenal metastases occur late in the course of disseminated cancers, and the primary site is usually already known.

Rarely metastatic adrenal tumors may lead to adrenal insufficiency, particularly in the context of large bilateral adrenal lesions (187). Therefore, in these circumstances, patients may present with symptoms and signs of adrenal insufficiency (fatigue, anorexia nausea, vomiting, postural hypotension, hyponatremia, and hyperkalemia). However, some of these clinical features are often difficult to distinguish from those of progressive metastatic malignancy.

##### Functional AI.

Ten to 15% of AIs secrete hormones in excess (170). The clinical features ascribed to functional AI will be discussed in “Endocrine work-up of AI.”

## Endocrine Work-up of AI

Clarification of the endocrine status of patients with AI is a cornerstone of management alongside the exclusion of malignancy. Clinical signs and symptoms of hormone excess and associated comorbidities together with a biochemical evaluation underpins personalized management. As a general principle, every patient with AI should be screened for adrenal catecholamine (although recent data has suggested this may not need to be the case in low Hounsfield unit lesions (188)) and glucocorticoid excess. Mineralocorticoid excess should be excluded in patients with hypertension and/or hypokalemia. Hirsutism or virilization or suspicion of an ACC should prompt measurement of androgens and gynecomastia estrogens.

### Screening for pheochromocytoma

Pheochromocytoma may present incidentally, but on closer questioning, approximately 50% of cases have classical paroxysmal symptoms of sweating, headache, and tachycardia (189). Additionally, the diagnosis may be suspected if there is a family history of a heritable disorder commonly associated with pheochromocytoma as discussed in “Etiology and clinical presentation of AI.” Hypertension is a common sign of pheochromocytoma, but 5% to 15% of patients may have normal blood pressure at presentation, particularly in those with incidentaloma or having familial screening where the tumor may be smaller and less functionally active at presentation (190). Paradoxically, tumors that secrete only epinephrine may cause hypotension (191). Headache occurs in up to 90% of symptomatic patients (190) with sweating in 60% to 70%. Other symptoms include tremors, weakness, palpitations, anxiety, constipation, visual blurring, hyperglycemia, polyuria, and polydipsia (192).

Catecholamine-induced cardiomyopathy related to pheochromocytoma may present with signs of heart failure such as pulmonary edema (193). Rarely patients may also present with “pheochromocytoma crisis,” with hypertension or hypotension, multiorgan failure, psychiatric disorder, and hyperthermia (194).

Patients with familial pheochromocytoma may be asymptomatic in 50% of cases, with only a third presenting with hypertension, possibly representing earlier diagnosis and smaller lesions as a result of regular screening in these patients (195,196). While evaluation for catecholamine excess is recommended by ESE/ENSAT guidelines in all AI patients (4), it may be possible to omit this in lipid-rich cortical adenomas. As discussed in detail in “Imaging evaluation of an AI,” an unenhanced/noncontrast CT is recommended as the first-line investigation to confirm if an AI is homogeneous and to calculate the attenuation value measured in Hounsfield units (HU). A HU ≤ 10 is consistent with a benign adrenal adenoma or other benign lesions (eg, myelolipomas, lipomas). Mean HU scores for pheochromocytoma are 30 to 35 (197) with only 0.5% of pheochromocytoma having an unenhanced CT attenuation of ≤10 HU (97,197,198). Buitenwerf et al. (197) reviewed the CT images of 222 histologically proven pheochromocytomas, yielding only a single tumor with an unenhanced attenuation of <10 HU. Canu et al. (188) identified 2 (0.5%) of 376 histologically proven pheochromocytomas with unenhanced CT attenuation value of exactly 10 HU, 99.5% (n = 374) had a HU > 10. Despite the inherent drawbacks of the retrospective study design which include variable radiological techniques and selection of the region of interest, unenhanced attenuation is a valuable tool to distinguish lipid poor adenomas from pheochromocytomas. Set against this are the inherent risks of a missed diagnosis and prolonged exposure to catecholamines and cardiovascular morbidity/mortality (199,200). Additional pointers for considering biochemical testing in a patient with an unenhanced CT attenuation of ≤10 HU for pheochromocytoma are elderly patients (4), heterogeneity, or evidence of tumor necrosis.

Biochemical testing for pheochromocytoma has an excellent negative predictive value of 0.99. As described above in “Anatomy and physiology of the adrenal gland,” measurement of the O-methylated metabolites of catecholamines is now the main focus for specific measurement in an era of advanced analytics employing liquid chromatography tandem mass spectrometry (LC-MS/MS) (201). Plasma free or 24-hour urine fractionated metanephrines are recommended as the screening test of choice for pheochromocytoma with a sensitivity of 90% to 95% (202, 203). Specificity rates of 85% to 89% decrease to 77% in elderly people and can be improved by using age-adjusted reference ranges (204-206). Similarly, false positives can occur with concomitant medications such as sympathomimetic drugs or interfering substances including caffeine; avoiding these for 24 hours before testing is recommended (108,207,208). For plasma free metanephrines, blood is drawn after resting in the supine position at least 30 minutes (209) into prechilled heparinized tubes (108, 210). For 24-hour urine estimation, the use of acidified containers (to achieve urine pH <4) and storage in a cold place is recommended; measurement of creatinine is often used to ensure adequate collection (108,202,211).

### Screening for autonomous cortisol secretion

The clinical features of Cushing’s syndrome are well defined; while individual susceptibility varies, generally, these are dictated by the degree and duration of cortisol hypersecretion. In many cases of AI with documented autonomous cortisol hypersecretion (ACS), cortisol secretion rates may not be significantly elevated. As a result, the patient may be asymptomatic, have no clinical features and have few comorbidities that may be ascribed to cortisol hypersecretion.

With nonspecific and wide-ranging signs and symptoms, diagnosing cortisol excess in patients with AI, particularly at levels that may be only marginally above “normal,” is challenging. For patients with overt Cushing’s syndrome, the Endocrine Society recommends the use of 2 of 3 highly sensitive screening tests: 24-hour urine free cortisol (UFC) excretion, late-night salivary cortisol (LNSC) levels and 9 am plasma cortisol following an overnight dexamethasone suppression test (1-mg dexamethasone suppression test [DST]; or low-dose DST) (212). Different assays (radioimmunoassay (RIA), enzyme-linked immunosorbent assay, automated chemiluminescence, high-performance liquid chromatography or mass spectrometry), patient comorbidities causing physiological hypercortisolism, and pseudo-Cushing’s syndrome are all confounding factors in the assessment of cortisol excess in patients with AI (212-214).

ACS is defined as an alteration of the HPA axis characterized by ACTH independent cortisol excess often without clinical signs and symptoms of overt Cushing’s syndrome. Nomenclature around this term has caused confusion with multiple names being given to this phenomenon including “subclinical Cushing’s syndrome,” “subclinical hypercortisolism,” and “preclinical Cushing’s syndrome” have all been used. However, we suggest the universal adoption of the term “autonomous cortisol secretion” as proposed by ESE/ENSAT (4). Despite the absence of florid signs and symptoms, ACS in patients AI has been associated with hypertension (215), insulin resistance (216), type 2 diabetes mellitus (217), obesity (218), metabolic syndrome (219), and increased mortality (75). ACS has emerged as the commonest functional abnormality in patients with AI with prevalence rates of up to 20% (64,65,72).

As highlighted through a summary of published guidelines, there is no gold standard test for diagnosing ACS. As can be seen in Table 5 there is a wide variety of first screening, secondary screening and confirmation tests depending on the guideline. The sensitivity and specificity of these tests in patients with ACS have also been widely reported and are summarized in Table 6. Overall, the consensus from these recommendations supports the use of the 1-mg DST as having the highest sensitivity for screening for ACS, Table 6. A post-dexamethasone cortisol level ≤1.8 µg/dL (≤50 nmol/L) is considered “normal” and excludes cortisol excess in most patients. Levels between 1.9 and 5.0 μg/dL (50-140 nmol/L) may indicate “possible autonomous cortisol secretion,” and cortisol levels above 5.0 μg/dL (140 nmol/L) are suggested to confirm ACS (79,220). Sensitivity increases as the stringency of the “cut-off” is lowered; from 80% at a cut off of 140 nmol/L to 95% at 50 nmol/L, but this is offset by lower specificity, such that at a cut off of 50 nmol/L 20% of the “normal” population will fail to suppress cortisol. Even at a cut-off concentration of 140 nmol/L specificity is only 95%; the investigator must consider that a false positive result is far more likely than a true positive (45). Furthermore, many of the age-related comorbidities ascribed to ACS (notably obesity, diabetes, anxiety/depression—see later) are in themselves causes of physiological hypercortisolism, and therefore confirming a diagnosis and ascribing clinical features to ACS that might prompt surgical intervention remains a major challenge.

**Table 5.** Summary of published guidelines for the diagnosis of subclinical Cushing’s syndrome (SCS)/autonomous cortisol secretion (ACS) in patients with AI.

Guideline (year) (ref.)	NIH (2003) (221)	Endocrine Society (2008) (212)	AACE/AAES (2009) (86)	FSE (2008) (7)	IACE (2011) (222)	ESE/ENSAT (2016) (4)	Korea (2017) (223)	JES (2018) (224)
Terminology	SCS			SCS		ACS	Asymptomatic hypercortisolism	SCS
Country	USA	USA	USA	France	Italy	Europe	Korea	Japan
**First screening test (cut-off)**								
1-mg DST cut-off point	Yes >5 μg/dL: possible SCS	Yes >1.8 μg/dL	Yes >5 μg/dL	Yes >1.8 µg/dL	Yes <1.8 μg/dL: exclude >5.0 μg/dL: consider 1.8–5.0 μg/dL: indeterminate	Yes <1.8 μg/dL: exclude >5.0 μg/dL: ACS 1.8–5.0 μg/dL: possible ACS	Yes <1.8 μg/dL: normal >5.0 μg/dL: asymptomatic hypercortisolism 1.8–5.0 μg/dL: additional testing	Yes >5.0 μg/dL ≥3 μg/dL with (any of A-D) or E ≥1.8 μg/dL with (A and B) or E
Late night salivary cortisol	NM	Yes (> 145 ng/dL)	**NR**	**NR**	**NR**	**NR**	NM	**NR**
**Second screening test**								
24-hour UFC	NM	**NR**	**NR**	Yes	Yes	Yes (if 1mg DST > 5ug/dL)	Not recommended	
Late-night serum cortisol	NM	NM	NM	Yes	Yes	NM	NM	Serum 21.00-24.00 ≥5 μg/dL
ACTH							Yes	
Late-night salivary cortisol	NM	NM	**NR**	NM	**NR**	Yes (if 1mg DST > 5ug/dL)	NM	
4-mg DST	NM	NM	NM	NM	**NR**	Yes (if 1mg DST > 5ug/dL)		**NR**
**Confirmatory test**								
4-mg DST	NM	**NR**	Yes	NM	**NR**	NM		**NR**
8-mg DST	NM	**NR**		NM	**NR**	NM		**NR**
**Localization (adrenal in origin)**								
ACTH	NM	Yes	Yes	Yes	Yes	Yes		<10 pg/mL
DHEA-S	NM	Yes	Yes	**NR**	**NR**	NM	NM	
4-mg DST	NM	NM	NM			NM	NM	
8-mg DST	NM	NM	NM			NM	NM	
Adrenal scintigraphy	**NR**	NM	NM	Yes	**NR**	NM	NM	

Abbreviations: NIH, National Institutes of Health; ES, Endocrine Society; AACE/AAES, American Association of Clinical Endocrinologists/American Association of Endocrine Surgeons; FSE, French Society of Endocrinology; IACE, Italian Association of Clinical Endocrinologists; DST, dexamethasone suppression test; ACTH, adrenocorticotrophic hormone; DHEAS, DHEA sulfate; NM, not mentioned; NR, not recommended; JES, Japan Endocrine Society; UFC, urine free cortisol.

JES abbreviations: A, low plasma levels of ACTH in the early morning; B, no diurnal changes in serum cortisol levels; C, unilateral uptake on adrenal scintigraphy; D, low serum levels of DHEA-S; E, transient adrenal insufficiency or atrophy of the attached normal adrenal cortex after removal of the adrenal tumor.

Adapted from reference (224).

**Table 6.** Summary of published data on diagnostic screening tests for investigating autonomous cortisol secretion and Cushing’s syndrome in patients with adrenal incidentaloma.

		CS					ACS or mild CS or occult CS				
Test	Cut-off	Sensitivity (%)	Specificity (%)	False positive (%)	False negative (%)	Ref.	Sensitivity (%)	Specificity (%)	PPV (%)	NPV (%)	Ref.
1-mg DST	1.8 µg/dL (50 nmol/L)	95-100	60-80.2	8	0	(225,226)	75-100	62–72	59	100	(64,68,227-231)
	2.0 µg/dL						91.3	56.3			(68,230,231)
	2.2 µg/dL						100	67			(230,231)
	3 µg/dL (86 nmol/L)	100	91.8			(226)	52.4-86.4	52.4-96.3			(230,231)
	5 µg/dL (138 nmol/L)	86.5	92-97.4	3	12-15	(225)	44–83.3	83-100	57	95	(64,68,227,228,230,231)
LNSC	2.7 nmol/L (RIA)	97	77			(232)					
	2.8 nmol/L (LC-MS/MS)						31.3	83.3			(233)
	100 ng/dL (2.76 nmol/L) LC-MS/MS	93	91			(234)	93	91			(234)
	0.350 μg/dL						93.8	79.5			(227)
	3.6 nmol/L (RIA)	92	100			(232)					
	5.1 nmol/L	100	100			(226)	22.7	87.7			(235)
	14.46 nmol/L						86 (73–93)	92 (86–95)			(228)
24-h UFC	80 µg (170 nmol) or ULN	91-96	89.6-92.5	8	2	(225, 226)	31.8-76	80-100	49 (100)	96 (93.3)	(227,228)
DHEA-S ratio	1.2						100	91.1			(236)
ACTH							41-86.4	38-96	47	96	(64,230)
Midnight serum cortisol	1.8 µg/dL (50 nmol/L)	99-100	20.2			(225)	22.7	87.7			(64)
	5 µg/dL (135 nmol/L)	95-100	73.9			(225)	77	68			
	7.5 µg/dL	90-96	87.7			(225)					
Plasma steroid metabolomic							Sensitivity 97 AUC 0.991				(237)
**Combination of tests**											
24-hour UFC + LNSC	>70 µg UFC + LNSC > 5.4 µg/dL (149 nmol/L)						30.8	100			
1-mg DST and 24-hour UFC and ACTH <10 pg/mL	>70 µg UFC + LNSC >3 µg/dL (86 nmol/L)						55.6-65.2	68.8-82.9			(230)
1-mg DST + Dexamethasone level > 3.3 nmol/L	Serum cortisol 1.8 µg/dL (50 nmol/L)						100	84			(238)
1-mg DST + Dexamethasone level > 3.3 nmol/L	Salivary cortisol 0.55 nmol/L						71	90			(238)
1-mg DST + dexamethasone level >3.3 nmol/L	Salivary cortisone 2.7 nmol/L						91	82			(238)
1 mg DST + LNSC (LC-MS/MS)	Serum cortisol 1.8 µg/dL (50 nmol/L), LNSC 1.4 nmol/L						88.9	85.2			(233)

Abbreviations: CS, Cushing’s syndrome; ACS, autonomous cortisol secretion; LRneg, negative likelihood ratio; LRpos, positive likelihood ratio; CI, confidence interval; 1-mg dexamethasone suppression test (DST); LNSC, late night salivary cortisol; PPV, positive predictive value [TP/(TP + FP)]; negative NPV, negative predictive value [TN/(TN + FN)]; DHEAS ratio calculated by dividing the measured DHEAS by the lower limit of the reference range (age and sex matched); ULN, upper limit of normal.

*Consider pseudo-CS state: alcoholism, psychiatric disorders, stress, diabetes, obesity, vigorous exercise, pain, hypothalamic amenorrhea, pregnancy.

UFC in isolation has a low sensitivity (31.8-76.0%) in diagnosing ACS but this can be improved by LC-MS/MS methodology (sensitivity 98%, specificity 91%, and negative/positive likelihood ratios of 0.02/10.83, respectively). A combination of the 1-mg DST with UFC decreases the false positive rate further (239). LNSC is emerging as perhaps the most accurate test in diagnosing overt Cushing’s syndrome (240), but for ACS the published studies have given mixed results. In some cases, sensitivity reached 93.8%, but with relatively poor specificity at 79.5% (227) (Table 6).

An Italian study diagnosed ACS based on 2 out of the following 3 tests: serum cortisol >3 µg/dL (83 nmol/L) after 1 mg of ONDST, UFC >70 µg/24 hours (193 nmol/24 hours) or ACTH <10 pg/mL (2.2 pmol/L) (228). While this was put forward as a way of improving accuracy, it also increases the likelihood of a false positive result and is not our usual practice. Intuitively a low/suppressed ACTH would seem a sensible adjunct in the diagnosis of ACS (Tables 5 and 6).

#### **Clinical consequences of ACS**—**cardiometabolic and bone.**

Based on the established clinical features of overt Cushing’s syndrome, there is now an extensive literature on the potential clinical impact of more modest increases in cortisol secretion as seen in ACS.

This has been reviewed extensively in focused articles on “subclinical Cushing’s” (now termed ACS) (241-245). In brief, cardiovascular morbidity includes increased left ventricular hypertrophy and arterial stiffness (246), hypertension, coronary heart disease, stroke, and fatal or nonfatal myocardial infarction. ACS is associated with mild to moderate hypertension in 41% to 92% of cases (47). A recent systematic review showed that ACS patients had a higher prevalence of cardiovascular events (16.7-26.8%) compared with patients with nonfunctional AI (6.7-10.4%) (79,244,245,247). Increased cardiovascular disease has been linked to the post-1-mg DST cortisol level as an independent cardiovascular risk factor (245). A study with up to 15 years of follow-up in patients with ACS has documented increased cardiovascular morbidity (43% vs 8.8%, *P* < .005) and mortality (22.6% vs 2.5%, *P* < .02) in ACS patients compared with nonfunctioning AI. Pulmonary infection and cardiovascular complications were the major causes of death (220,247).

It is well established that glucose intolerance and diabetes mellitus are prevalent features of overt Cushing’s syndrome (228,248). Impaired glucose tolerance or diabetes has been reported to occur in 10% to 69% of patients with ACS (249) and improved glycemic control (and in some cases reversal of diabetes) following surgery is reported in some studies but not others (Table 7).

**Table 7.** Summary of published studies evaluating the effect of adrenalectomy on clinical and biochemical features of autonomous cortisol secretion.

				Proportion of patients with improvement postop		
Study, year (ref.)	Design	Diagnostic criteria DST cortisol nmol/L ACTH pg/mL	Operated patients (n)	Arterial HTN	Weight	Impaired glucose metabolism
Perysinakis 2013 (250)	Retrospective no control	DST >50 and 1 of the following: ACTH <2.2 24-h UFC >276 blunted diurnal CCR	29	12/17	6/14	5/12
Iacobone 2012 (251)	Retrospective	DST >138 ACTH <2.2 high 24-h UFC	20	8/15	6/15	5/10
Maehana 2012 (252)	Retrospective nonfunctioning No control	DST >50 and 1 of the following: low ACTH, loss of CCR, scintigraphy, low DHEAS	13	5/7	n/a	2/2
Guerrieri 2010 (253)	Retrospective	2 of the following: DST >50, high 24-h UFC ACTH <2.2.	19	12/12	n/a	n/a
Chiodini 2010 (254)	Retrospective	2 of: the following DST >83, high 24-h UFC, ACTH <2.2.	25	n/a	n/a	n/a
Toniato 2009 (255)	Prospective randomized	DST >69 and 2 of the following: low ACTH, high 24-h UFC, loss of CCR, low DHEAS	23	12/18	3/6	5/8
Tsuiki 2008 (243)	Retrospective	DST (8mg) >28 and one of the following: ACTH<2.2, blunted ACTH after CRH, loss of CCR, low DHEAS, scintigraphy	10	5/6	0/3	2/9
Salcuni 2016 (256)	Retrospective 23 controls refused surgery	DST >138 or 2 of the following DST >83 low ACTH high 24h-UFC	32	No change	—	No change

Adapted from Iacobone et al. (257).

Abbreviations: n/a, not assessed/ available; DST, dexamethasone suppression test; ACTH, adrenocorticotropic hormone; UFC, urinary free cortisol; CCR, cortisol circadian rhythm (ratio of plasma cortisol at 24.00 hours to 08.00 hours >50%); DHEAS, dehydroepiandrosterone sulfate; CRH, corticotropin-releasing hormone.

Similarly, the deleterious effects of chronic glucocorticoid excess on bone mineral density leading to osteoporosis are well established (258). However, in patients with ACS, a mixed picture is reported with either decreased, normal, or even increased bone formation reported alongside normal bone resorption (229,242,259-262). This variance is likely to be explained by differences in bone turnover markers studied, underlying vitamin D, parathyroid hormone concentrations, age, sex (notably the percentage of postmenopausal women), and the sample size. Cortisol affects trabecular bone to a greater degree than cortical bone, which is similar to the postmenopausal state. Both trabecular (65,261-263) and cortical (259,262,264-266) bone loss have been described in patients with ACS. For bone fracture, a meta-analysis reported a prevalence of vertebral fracture in patients with ACS of 63.6% (95% CI 55.98-71.26%) (254) and this risk is independent of age, sex, gonadal status, or bone mineral density (264).

#### Reversal of ACS.

Several studies have reported the outcome of surgical intervention but most involve small numbers and are retrospective, uncontrolled, and lack randomization (230,243,250-253,255) (Table 7). ClinicalTrials.gov lists several prospective trials that are underway but are yet to report.

Metabolic improvement after adrenalectomy, including weight loss, blood pressure lowering, glucose tolerance, lower lipids, and beneficial effects on bone have been reported (230,267). A recent systematic review addressed cardiovascular benefit in ACS patients (247) with adrenalectomy improving cardiovascular outcome and mortality (4,247).

In the absence of a robust evidence base for the surgical treatment of ACS and reversal of symptoms and signs, establishing a causative link of ACS to cardiometabolic traits that increase naturally with age and low bone mineral density remains a challenge. It is reasonable to assume that patients, albeit with mild forms of adrenal Cushing’s syndrome, should benefit from surgery, noting that even in patients with established Cushing’s syndrome, the reversal of the clinical phenotype can take many months or years. The degree of hypercortisolism within the ACS definition, the severity of clinical features detailed earlier, balanced against risks of surgery in an aged population must be considered as an individualized approach. The real risk is an unnecessary procedure for an elderly patient with coincidental age-related comorbidities that have no causative relationship to a false positive ACS diagnosis. Over and above evidence-based guidelines for the non-AI background population, we strongly recommend aggressive treatment of comorbidities (hypertension, hyperlipidemia, glucose intolerance, weight, and reduced bone mass) in patients with AI. This will likely have a far greater impact on preventing and reversing cardiometabolic morbidity and mortality than lowering borderline cortisol secretion rates. Large randomized prospective trials of treatment of ACS are required to answer many of the above questions.

#### The natural history of “untreated” ACS.

A recent New Zealand study, prospectively evaluated 101 patients with benign adrenal adenomas over a 3-year follow up period. Nine patients had evidence of ACS at diagnosis (defined as an elevated 24-hour UFC and a 1mg-DST), while the remaining 92 patients were diagnosed with a nonfunctioning AI. At 3 years, 5 of the 9 patients with ACS at diagnosis (44%) showed normalization of cortisol parameters, while 5 of the 92 patients with a nonfunctional AI developed ACS (5%) (268). Barzon et al. reported the cumulative incidence of ACS after 1and 5 years as 3.8% and 6.6%, respectively (68). An Italian group reported an increased risk of ACS development if the adenoma size was >2.4 cm (sensitivity 73.3% and specificity 60.5%, *P* = .014) (244).

A recent systematic review and meta-analysis reported the follow-up of 4121 patients with nonfunctioning adrenal tumors (NFATs) and/or mild autonomous cortisol excess (MACE) (269). Clinically overt hormone excess (Cushing’s syndrome) was unlikely to develop (<0.1%) in patients with NFATs or MACE. Only 4.3% of patients with NFATs developed MACE, and pre-existing MACE was unlikely to resolve (<0.1%). Hypertension, obesity, dyslipidemia, and type 2 diabetes were more likely to develop and worsen in MACE than NFATs and new cardiovascular events were more prevalent in MACE (15.5%) than NFATs (6.4%) during follow-up. However, reported mortality (11.2%) was similar between NFATs and MACE.

Guidelines suggest that where functional secretion has been excluded at diagnosis, repeat endocrine testing is not required unless clinical features significantly change (4). Annual clinical assessment for up to 5 years is recommended in patients with ACS (4) together with an ongoing individualized risk assessment for potential benefit from surgical intervention based upon strengthening evidence linking cortisol excess to clinical symptoms and signs.

### Screening for primary aldosteronism

The discovery of hyperaldosteronism in the context of an AI is relatively uncommon, compared with the detection of cortisol hypersecretion or pheochromocytoma (45,270). Normokalemic primary aldosteronism (PA) has been reported in up to 5.5% of AI (271). A few publications have reported normotensive PA in AI; however, most of these patients demonstrated occasional hypokalemia (8,272-274). Hypertension and hypokalemia are the major classical clinical findings of PA and the presence of either in the context of AI should prompt appropriate screening investigations. PA is associated with low plasma renin activity (or concentration) because aldosterone-induced hypervolemia results in suppression of renin release (275). Although hypokalemia in the context of hypertension in a patient with AI is suspicious of PA, the finding of hypokalemia is not a reliable marker. A recent multicenter analysis estimated that only 9% to 37% of patients with primary hyperaldosteronism were hypokalemic (276,277).

PA is associated with an increased risk of stroke (odds ratio [OR] 2.58, 95% CI 1.93-3.45), coronary artery disease (1.77, 1.10-2.83), atrial fibrillation (3.52, 2.06-5.99), heart failure (2.05, 1.11-3.78), and mortality (hazard ratio [HR] 1.34, 95% CI 1.06-1.71) compared with matched populations with essential hypertension (278,279). Therefore, the diagnosis of PA is important and requires either surgical excision or targeted antihypertensive therapy with mineralocorticoid receptor antagonists. Diabetes mellitus/metabolic syndrome (278,279), renal damage (280), impaired bone mineral density (81), and osteoporosis (264,281,282) have also been reported to be more prevalent in AI patients with PA.

Clinical guidelines recommend screening for PA in every (AI) patient with hypertension or hypokalemia (283,284). However, normotensive patients with suppressible aldosterone/renin ratios may frequently develop hypertension and PA (285). Brown and colleagues (285) reported that populations with suppressed plasma renin activity (≤0.5 ng/mL/h) and high aldosterone levels were at increased risk of hypertension (HR 1.18; 95% CI 1.03-1.36) (285). These studies draw attention to the concept of a preclinical stage of PA. Further studies are required before the routine screening of all AI patients is recommended.

The 3-step approach for screening, confirmatory, and subtype classification of PA has been thoroughly covered in an excellent Endocrine Society Clinical Practice Guideline and will not be repeated here (284). Only to say that initial assessment using plasma aldosterone concentrations and plasma renin activity or the direct renin concentration and derivation of the aldosterone/renin ratios yields a sensitivity of 68% to 94% and negative predictive value approaching 100%.

### AI-secreting sex steroids

AIs that secrete either estrogen or testosterone in isolation are rare. Although benign cortisol-secreting adenomas may very rarely produce androgens (286), elevated sex steroids are highly suspicious for ACC and urgent diagnostic evaluation is required (287-289).

In women, androgen-secreting tumors may present with features of virilization, such as excessive facial hair growth, skin changes such as acne, deepening of the voice, clitoromegaly, and male pattern baldness. Estrogen-secreting tumors in women may cause irregular uterine bleeding and breast tenderness. In men, estrogen-secreting tumors may cause feminization with decreased libido, testicular atrophy, and gynecomastia as key clinical features.

## Imaging Evaluation of an AI

Multiple different groups have developed guidelines for the work-up of AIs (4). A key consideration for the diagnostic workup of an AI is whether there is a possibility of malignancy. Patients with an AI >1 cm in the short axis should undergo an imaging procedure to determine if the lesion is benign or malignant at the time of initial diagnosis. Some lesions with overtly benign features such as the presence of bulk macroscopic fat (myelolipoma) or simple cysts may not require any further imaging assessment.

Multiple imaging parameters are employed for the differential diagnosis of AI. There is a correlation between tumor size and risk of adrenocortical cancer: 2% risk in AIs <4 cm, 6% in AIs 4.1 to 6 cm, and 25% in AIs >6 cm (290). A large Italian study of patients with AI (n = 887) reported that 90% of ACCs had a diameter of >4 cm at presentation, a 4-cm cut-off had a 93% sensitivity for detecting ACCs (64, 137). Other imaging features used for characterization include unenhanced CT with the assessment of tumor density, contrast-enhanced timed washout CT studies, MRI chemical shift analysis, and, more recently, 18-fluorodeoxyglucose (FDG) positron emission tomography (FDG-PET) in combination with CT (PET-CT) (291).

### Unenhanced CT

**Figure 6.**
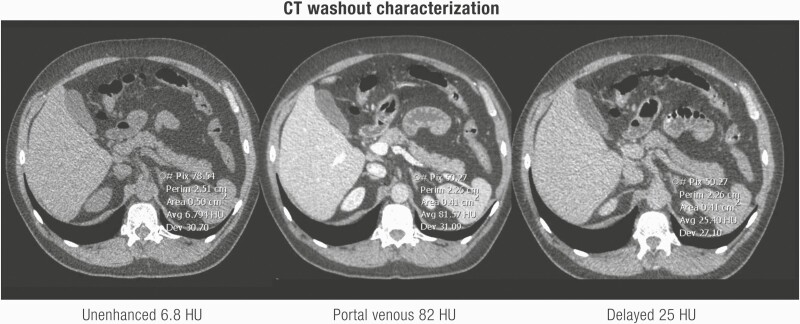
Characterization of AI by CT. Axial images obtained pre and post-IV contrast at the portal-venous (60-70 seconds) and delayed (10-15 minutes) phases post injection. Calculation of adrenal lesion attenuation value showing benign pattern washout in a lipid-rich left adrenal adenoma.

For all AIs, a noncontrast (unenhanced) CT is recommended as the first-line investigation. HU assessment on an unenhanced CT is a method for quantifying X-ray absorption of tissues compared with water, which conventionally has a HU of 0 (Fig. 6). Care is required when placing a region of interest to measure HU, this should cover at least two-thirds of the circumference of the nodule while avoiding areas of heterogeneity such as necrosis or calcification (292,293). A recent meta-analysis (291) on the use of imaging for differentiating benign from malignant adrenal lesions identified only 2 studies with 102 true incidentalomas, based on strict meta-analysis inclusion criteria. In patients without known extra-adrenal malignancy, an HU of ≤10 on a noncontrast CT is consistent with a lipid-rich benign adenoma (294). Importantly, the assessment of HU is for homogeneous lesions and care must be taken in heterogeneous lesions given the variability of HU and the potential to miss more solid components that may be malignant.

### Contrast-enhanced washout CT

Approximately 30% of benign adrenal adenomas do not contain large amounts of fat and have an attenuation value of >10 HU (ie, lipid-poor adenomas), these lesions cannot be reliably characterized on unenhanced CT due to an overlapping density with malignant lesions and pheochromocytomas (295,296). For lesions with an attenuation value of >10 HU, further assessment is required and there is no clear consensus regarding which is the best second-line imaging for these patients. Our institutional practice is to perform a dedicated adrenal washout protocol CT as the next test because this is more accurate for characterizing lipid-poor adenomas than MRI (297), although as a recent meta-analysis has shown there is a need for further studies to optimize this technique as there is significant variability in scanning protocols and timings described and sensitivities and specificities (291).

Washout studies consist of CT imaging performed before and at 2 time points after administration of intravenous contrast (typically, imaging is performed at 60-90 seconds and 10 or 15 minutes postcontrast) with no clear evidence about the best time interval for the later time point (298). Adenomas typically enhance rapidly and show prompt washout of intravenous contrast, in contrast to malignant adrenal lesions which usually enhance rapidly but demonstrate a slower washout of contrast medium. Absolute washout values of >60% (early enhanced HU – delayed HU)/(early enhanced HU – unenhanced HU) × 100% suggest a benign adenoma (295,299). Relative washout can be calculated when unenhanced CT is not available (early enhanced HU – delayed HU)/enhanced HU × 100%. Values of > 40% suggest a benign adenoma (296,299,300) (Fig. 6). However, only 1 previous study has evaluated the test performance of CT contrast-enhanced washout in the evaluation of truly incidentally discovered adrenal masses (301), that is in patients not undergoing surveillance imaging for previous nonadrenal malignancy. This study reported high sensitivity (96%) and specificity (95%) for differentiating benign from malignant adrenal lesions (301); however, it considered only 50 cases, therefore larger prospective studies are required to validate the performance of contrast CT washout in patients with AI. Caution is also required because whilst pheochromocytomas usually show slow contrast washout, occasionally they can mimic benign lipid-poor adenomas by showing rapid washout (302). Therefore, following multidisciplinary team (MDT) discussion if there is still a suspicion regarding a lesion with indeterminate HU further scanning should be requested to ensure no further growth.

### Dual-energy CT

Dual- or multi-energy CT is becoming more widely available and can provide additional specific data about attenuation properties of different materials at different energies (303). A frequent clinical dilemma is the detection of an AI on a single-phase contrast-enhanced CT typically performed 60 to 90 seconds postcontrast administration when the HU value of benign adenomas and nonadenoma lesions overlap significantly (304). Dual-energy CT offers the potential to extract a virtual noncontrast CT dataset from routinely acquired variable energy single-phase contrast-enhanced CT (305). A recent systematic review and meta-analysis reported that virtual non-contrast CT images generated from dual-energy CT demonstrate comparable sensitivity with standard noncontrast CT for diagnosis of lipid-rich adenomas, but data were only available from a small number of heterogeneous studies at high risk of bias (306). This technique requires prospective evaluation in a larger patient cohort before routine clinical use.

### Magnetic resonance imaging

**Figure 7.**
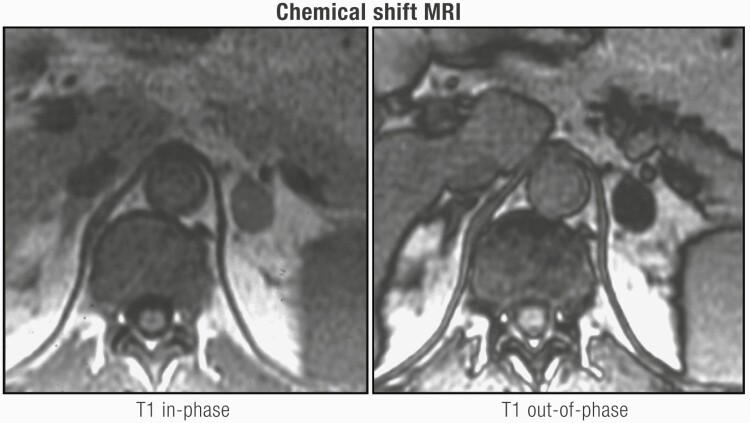
Chemical shift MRI. Paired axial T1-weighted MRI images showing loss of signal within a left adrenal nodule on out-of-phase imaging consistent with lipid-content within a lipid-rich adrenal adenoma.

More recent guidelines suggest that MRI evaluation of adrenal lesions should be used primarily as a problem-solving tool. MRI with chemical shift imaging should only be the first choice where CT is less desirable, for example during pregnancy, in children, or for patients with allergies to iodinated contrast (this is not an issue in noncontrast scans) (307). The use of chemical shift MRI to detect intracellular fat within lipid-rich adrenal adenomas and differentiate these from other lesions was first described in 1992 (310) (Fig. 7). There are both qualitative (visual analysis) and quantitative methods of imaging evaluation which involve assessing change in signal intensity of adrenal lesions between in-phase and out-of-phase sequences (309). A recent systematic review and meta-analysis reported that visual analysis and quantitative analysis of adrenal signal intensity index and adrenal-to-spleen ratio all have high accuracy for lipid-rich adenoma detection (95-98%), and diagnostic performance is not significantly improved by adrenal signal intensity index or the adrenal-to-spleen ratio (310). Consequently, if AIs are detected on MRI and findings are unambiguous for a benign lipid-rich adenoma further imaging may not be warranted.

### Positron emission tomography-computed tomography

**Figure 8.**
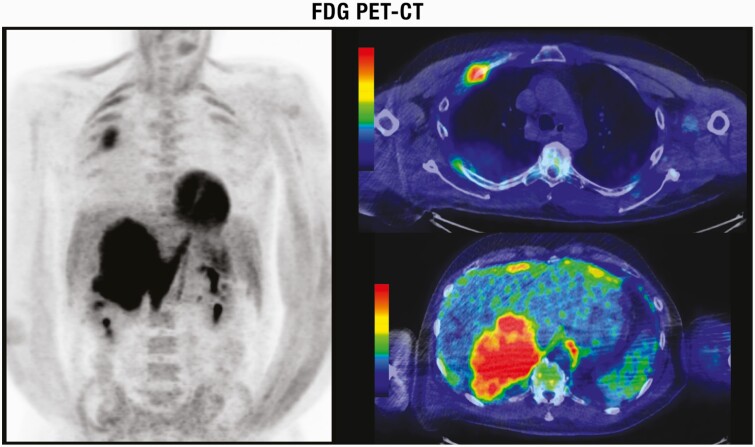
^18^F-fluorodeoxyglucose PET-CT. Coronal PET maximum intensity projection and axial fused PET-CT images in a patient with a locally advanced right adrenocortical carcinoma and an unsuspected tracer-avid right-sided rib metastasis.

FDG PET-CT has high diagnostic accuracy for characterization of adrenal masses with a pooled sensitivity of 91% and specificity of 91% in a recent meta-analysis of 29 studies involving 2421 patients (311). This technique is more expensive and less available than CT and MRI but may help to discriminate metastatic disease from benign masses in patients with known malignancy (4,312) (Fig. 8). One limitation is that benign adrenal lesions particularly functional adenomas and nonmetastatic pheochromocytomas can be FDG positive (313,314). As with MRI, there are both qualitative (visual analysis) and quantitative criteria used for the diagnosis of malignant adrenal lesions. Visual assessment of FDG activity within adrenal lesions compared to background (physiological) uptake in the liver or blood pool has similar pooled sensitivity and specificity (91-92%) to quantitative analysis using adrenal to liver standardized uptake value (SUV) ratio (89-91%) or maximum SUV (SUV_max_) (85-91%) (309). False-negative findings are recognized in the setting of small malignant lesions, metastases from non-FDG avid tumors such as some subtypes of renal carcinoma and well-differentiated neuroendocrine malignancies and in necrotic lesions (315). In patients with a suspected solitary adrenal metastasis being considered for adrenalectomy, FDG PET-CT should be considered to exclude extra-adrenal metastatic disease not detected on CT or MRI (4).

### Follow-up imaging

European guidelines suggest no further imaging for patients with adrenal lesions with benign characteristics <4 cm in diameter (4). This is further supported by a recent systematic review and meta-analysis of nonfunctioning adrenal lesions where a mean tumor growth of 2 mm occurred over a median of 52.8 months, and clinically significant tumor enlargement (≥10 mm) occurred in only 2.5% of patients, and none developed adrenal cancer (269).

Data from a large Italian cohort study reported a 4 cm size cut-off had the highest sensitivity (93%) but low specificity (24%) for prediction of malignancy (66). For this reason, larger lesions with clear benign features warrant interval reassessment with imaging after 6 to 12 months to exclude significant growth (>20% increase in size with at least 5 mm absolute increase in diameter) which would prompt surgical resection. Less marked interim lesion growth warrants reassessment with a further scan after 6 to 12 months.

Ideally, every patient with an AI should be managed by an expert MDT, incorporating an endocrinologist, radiologist, pathologist, and a surgeon, with significant expertise in adrenal tumors. However, this is a challenging aspiration given the increasing prevalence of AI. Therefore the ESE/ENSAT guideline (4) identifies certain patients who are most likely to benefit from MDT discussion. This includes conditions where one of the following criteria is met; imaging is not consistent with a benign lesion; there is evidence of hormone excess (including ACS); there is evidence of significant tumor growth during follow-up imaging; and where adrenal surgery is considered.

## Special Circumstances

### Bilateral adrenal incidentalomas

Bilateral adrenal masses may represent co-occurrence of different entities; therefore both lesions should be separately assessed as per recommendations for a unilateral lesion (4). Interestingly, in patients with bilateral disease, it is possible for 1 mass to be a nonfunctioning cortical adenoma while the other mass may be hormone secreting (316). Additionally, there is a possibility that both masses (particularly in the context of hemorrhage and metastases) may lead to adrenal hypofunction and therefore this should be considered in the evaluation of the patient with bilateral AIs.

#### Bilateral AI and ACS.

ACS is more frequently encountered in patients with bilateral AI than in those with unilateral lesions (317-319). A prospective study of 298 patients (224 with unilateral AI and 74 with bilateral AI) reported evidence of ACS in 35.1% of patients with bilateral AI, but only 17.9% of patients with unilateral AI (*P* = .003) (319). This supports previous retrospective studies (320-322) and a more recent surgical series including 112 unilateral AI and 23 bilateral AI, where mild cortisol excess was present in 21.7% bilateral AI versus 6.2% of unilateral AI (321). Vassiliadi et al. (320) demonstrated an exaggerated response of cortisol and ACTH in patients with bilateral AI during the combined tests of a DST followed by CRH stimulation when compared with unilateral AI and to a control group suggesting that regulation of the hypothalamic–pituitary axis was disturbed in patients with bilateral AI; however the diagnosis of BMAH was not excluded in this group (319).

PBMAH is suspected when there are bilateral AI and evidence of ACS (323). Cortisol secretion is in part regulated by the expression of multiple aberrant G protein-coupled receptors in the zona fasciculata cells including receptors for vasopressin, serotonin, luteinizing hormone/human chorionic gonadotropin, β -adrenergic agonists, gastric inhibitory polypeptide, glucagon, and angiotensin (324). These G protein-coupled receptors lead to activation of cAMP–PKA signaling pathway, similar to the pathway activated by ACTH receptor consequently leading to the transcription of steroidogenic factors.

On unenhanced CT, both adrenal glands are enlarged usually with multiple nodules but diffuse enlargement without nodules is also seen (325). On contrast-enhanced imaging, nodules can have marked enhancement, and HU >10 has also been described. Despite PBMAH being a benign disease limited studies using FDG uptake demonstrated an uptake similar to that seen in malignant tumors (326).

#### Bilateral AI and CAH.

CAH can rarely present as bilateral adrenal lesions in adult life, although the majority of patients present with symptoms of adrenal insufficiency in childhood. The most common form of CAH, affecting over 90% of individuals, involves the gene for 21-hydroxylase and if suspected 17-hydroxyprogesterone (17-OHP) testing and mutation analysis for the gene *CYP21B* should be undertaken. The measurement of 17-OHP in the setting of bilateral adrenal hyperplasia should be interpreted with caution. In some cases, increased levels of 17-OHP may represent increased secretion of steroid precursors (327) from the lesion(s) especially in malignant tumors or in bilateral macronodular adrenal hyperplasia. In these cases, low or suppressed ACTH levels may argue against CAH.

#### Bilateral AI and pheochromocytoma.

Pheochromocytomas occur bilaterally in 10% cases and although some cases are sporadic, they are often seen as part of a genetic syndrome, discussed in detail in “Etiology: Tumors of the Adrenal Medulla.” It should be noted that patients who have a unilateral pheochromocytoma may also have an incidental adrenal adenoma and as such every adrenal lesion should be assessed individually.

#### Bilateral AI and metastases.

The most common primary cancers to metastasize to the adrenal gland include lung, breast, renal, melanoma, and the gastrointestinal tract, although multiple other primary sites have been reported. The adrenal glands have been reported to be the fourth most common site of metastases in malignancy. In up to 75% of patients with an AI and extra-adrenal cancer, the adrenal mass is a metastasis and bilateral adrenal metastases are seen in a significant number of patients. However, 25% of patients with extra-adrenal cancer will have a primary adrenal lesion (101).

From an endocrine perspective, bilateral adrenal metastases may rarely cause adrenal insufficiency and patients should be clinically assessed and undergo further investigations if suspected. A unilateral adrenal metastasis would not cause adrenal insufficiency and patients do not usually require evaluation.

#### Management of bilateral AI.

Surgical management of unilateral AI is discussed in “Surgical Management of Adrenal Incidentaloma.” The management of bilateral AI is challenging because the size of the tumors is not a criterion for surgery. As there is no clear consensus on the criteria defining abnormal cortisol secretion, the indication for adrenalectomy in the case of bilateral adrenal lesions remains controversial. Bilateral adrenalectomy may be considered if urinary cortisol levels are higher than 3 to 4 times the upper limit of normal with both adrenals having a relatively symmetrical size (324). The European Society of Endocrinology suggests that in selected patients, unilateral adrenalectomy of the dominant lesion may be considered based on the age of the patient, degree of cortisol excess, general condition, comorbidities, and patient preference (4). A recently published observational study analyzed the long-term clinical and biochemical outcomes of unilateral adrenalectomy versus bilateral adrenalectomy in patients with PBMAH and ACS and compared it to the outcome of cortisol-producing adenomas treated with unilateral adrenalectomy (328). The data, albeit retrospective and observational, showed that unilateral adrenalectomy of patients with PMAH lead to initial clinical remission in 84% of patients but at follow-up (median, 50 months), only 32% of patients were biochemically controlled (defined as a cortisol after dexamethasone <1.8 μg/dL) compared with all patients with bilateral adrenalectomy and unilateral adrenalectomy for a cortisol-producing adenoma. Additionally, 3 deaths occurred in the group of patients who were not biochemically controlled after unilateral adrenalectomy.

Bilateral adrenalectomy is recommended for bilateral pheochromocytomas; however, the risk of adrenal insufficiency and the side effects of life long glucocorticoid replacement have led to certain centers performing adrenal cortex sparing resections particularly in the setting of genetic syndromes such as VHL and MEN2A where the risk of malignant tumors is low (325).

### Adrenal gland biopsy

The number of adrenal biopsies has significantly reduced in recent years due to improvements in noninvasive imaging techniques, which can increasingly accurately diagnose benign adrenal lesions. There are a number of reasons not to perform a biopsy on an adrenal lesion which includes the risk of seeding of adrenal cancer after biopsy (329), the adverse hemodynamic effects of performing a biopsy on an undiagnosed pheochromocytoma or paraganglioma, and most importantly of all it is frequently not possible to distinguish between an adrenal adenoma and an adrenal cortical carcinoma on the sample which is obtained and therefore it often does not help the clinical decision-making process.

In a recent systematic review and meta-analysis, Bancos et al. concluded that an adrenal biopsy is most useful in confirming the diagnosis of metastasis from extra-adrenal cancer (330). It should only be undertaken following detailed discussion at an adrenal tumor multidisciplinary meeting and if the lesion is not conclusively benign on imaging and, most importantly, the outcome will affect the therapeutic management of the patient (eg, if the patient has an extra-adrenal primary and a lesion in the adrenal would affect the staging and further management of the patient). Pheochromocytoma should be excluded before an adrenal biopsy, to prevent precipitating a potential hypertensive crisis. The biopsy itself is not without risk. The most frequent complications are pneumothorax (due to the proximity of the adrenal gland to the diaphragm) and hemorrhage. Patients should, therefore, be able to hold their breath and have normal coagulation (331).

## Surgical Management of Adrenal Incidentaloma

### Indications for unilateral adrenalectomy

The principle threat of a unilateral adrenal lesion, with indeterminate imaging characteristics, is an ACC. For ACC without metastases, surgery is the most important single therapeutic measure. Guidelines recommend that surgery for patients with ACC should be limited to referral centers and performed by surgeons with expertise in adrenal surgery (open and laparoscopic) and with a volume of more than 15 adrenalectomies per year (benign and malignant) (332).

Patients with a functional unilateral adenoma, with clinically significant hormone excess, are offered unilateral adrenalectomy. Patients with a unilateral adrenal adenoma and ACS, are considered for surgery on an individual basis and after MDT discussion (4). Surgery is taken into consideration along with the patient’s age, degree of cortisol excess, general health, comorbidities, and patient preference. In all patients, considered for surgery, ACTH-independent cortisol excess should be confirmed (4).

There is no absolute size for the recommendation of surgery in nonfunctioning adenomas. Due to the paucity of follow-up data on the natural history of large suspected benign AIs, and the realization that the larger the mass, the higher the incidence of malignancy (333), surgery may be considered in larger lesions (>4 cm) even if imaging characteristics suggest a benign lesion. Any indeterminate nodule on imaging greater than 4 cm should be offered surgery (4).

Occasionally myelolipomas and adrenal cysts are removed if they get to a significant size and cause compressive symptoms. Adrenal metastases are resected if appropriate in the context of the underlying pathology. In general, patients are deemed suitable if the underlying extra-adrenal malignancy is controlled, the metastasis is isolated to the adrenal gland and the patient has an adequate performance status to justify aggressive treatment.

### Perioperative care of patients for adrenalectomy

Adrenal surgery involves a multidisciplinary team approach in order to achieve the best outcomes. Any patient who has unilateral adrenalectomy for Cushing’s syndrome or evidence of ACS is at risk of contralateral adrenal gland suppression, due to the chronically elevated pre-operative cortisol levels with consequent ACTH suppression and adrenal atrophy. Peri- and postoperative stress dose glucocorticoids are required, which are weaned down to oral maintenance therapy prior to discharge. After unilateral adrenalectomy, patients require regular follow-up and synacthen testing to assess when the contralateral adrenal function recovers and steroid replacement can be stopped. All patients require education regarding hydrocortisone treatment prior to discharge and an understanding of adrenal insufficiency sick rules.

Before adrenal surgery for pheochromocytoma patients are treated with α -blockers, intravascular volume expansion, and in some patients β -blockers (particularly if concerns regarding tachyarrhythmia are present) (180). After the tumor is devascularized, especially after dividing the adrenal vein intraoperatively, patients may develop profound hypotension, which requires fluid optimization and inotropic support, which may need to be continued in the initial postoperative period (334).

Patients with AI and primary hyperaldosteronism are often prescribed a mineralocorticoid receptor antagonist, such as spironolactone or eplerenone. Following adrenalectomy for PA, this should be discontinued to prevent hyperkalemia. Other antihypertensives are stopped or reduced depending on the patient’s postoperative blood pressure. Older patients and those with longstanding hypertension are more likely to have irreversible vascular damage and have persistent hypertension requiring antihypertensive medication, despite successful surgery (335).

### Surgical techniques

Adrenal surgery has significantly changed over the past 100 years. There have been multiple open approaches to the adrenal gland, including transperitoneal, posterior, and flank. In 1992 the first laparoscopic transperitoneal adrenalectomy was performed and more recently the laparoscopic posterior (retroperitoneoscopic) adrenalectomy technique has been developed.

Laparoscopic adrenalectomy is now considered the gold standard of surgical treatment for the majority of small and medium-sized adrenal lesions. Multiple studies have demonstrated a shorter hospital stay, reduced pain, minimal morbidity, and improved recovery (336). The adrenal is a relatively inaccessible organ and difficult to access through an open incision, therefore lending itself to minimally invasive surgery. The posterior retroperitoneoscopic adrenalectomy has been developed more recently and uses high insufflation pressures in the retroperitoneal cavity. It provides direct access to the adrenal gland, avoids the intra-abdominal cavity and allows bilateral procedures to take place without repositioning the patient. Disadvantages of the posterior retroperitoneoscopic approach include a smaller working space, which makes removal of larger tumors more challenging and increased difficulty in patients with a high body mass index (BMI). This technique has been reported to lead to reduced post-operative analgesia and length of hospital stay (337,338).

More recently robotic-assisted laparoscopic adrenalectomy has been developed via the transperitoneal technique and has been shown to be safe and effective. Robotic surgery offers a greater range of motion, improved magnification, and stereoscopic vision than traditional laparoscopic surgery. The main advantages have been reported in patients with a high BMI and larger tumors. However, there is an increased cost, longer operative time, and a significant learning curve (339).

The role of laparoscopic surgery for the treatment of ACCs remains controversial. Complete resection (R0) of the entire tumor along with any adjacent involved structures or lymph nodes is the only chance of cure. Laparoscopic surgery is only advised if the tumor can be removed completely with an intact adrenal capsule and there is no evidence of tumor invasion. Based on the available data and clinical experience, the European guidelines suggest that laparoscopic adrenalectomy may be justified for adrenal tumors ≤6 cm with radiological signs of malignancy and without evidence of local invasion (4). Open resection is recommended in larger tumors due to the higher risk of tumor capsule rupture and in those with any evidence of local invasion and or when adjacent organ resection is required. The decision to convert to an open procedure should be made early, before any rupture of the tumor capsule (340). In many cases, an individualized decision process by a multidisciplinary team is required to find the best surgical approach for a given patient.

Solitary adrenal metastases often are confined within the adrenal gland and laparoscopic adrenalectomy is a safe technique, which achieves negative margins and reduces patient morbidity (340). If there is any evidence of local invasion into surrounding tissue intraoperatively the procedure should be converted to an open operation.

### Follow-up in patients without surgery

The American Association of Clinical Endocrinologists recommends repeated imaging for up to 5 years for benign tumors (86). However, the more recent European guidelines do not recommend further imaging for benign, nonfunctioning lesions of less than 4 cm (4). Patients with adrenal lesions over 4cm or indeterminate lesions, who have not undergone surgery are recommended to have repeat imaging, either a noncontrast CT or MRI in 6 to 12 months, the optimal timing is at the discretion of the MDT and depends on the index of suspicion (4). Surgical resection is recommended if there is a 20% increase in size, in addition to at least a 5 mm increase in diameter over this period. Both adrenocortical cancers and metastases usually demonstrate rapid growth over months, in contrast to a benign adenoma. Further imaging should be undertaken, again at 6 to 12 months, if there is an increase in size, but not at the threshold for surgical intervention (4). For younger patients, MRI surveillance, rather than CT, is preferred due to lower radiation exposure. In elderly patients follow up with imaging, even with nodules over 4 cm might be favored over surgery, in the presence of multiple comorbidities. Patients are treated on an individual basis but it is acknowledged that many clinicians and patients would not feel comfortable following up a tumor over 4 cm on imaging, even if radiologically benign, in a young patient and surgery is often the preferred option.

Repeat hormonal testing is not advised in patients without evidence of hormone oversecretion on their initial assessment (4). It should only be considered if patients develop new clinical signs of adrenal hormone hypersecretion or worsening of comorbidities, including diabetes, hypertension, or osteoporosis (4).

The risk of patients with ACS progressing to overt Cushing’s syndrome is negligible; however, ACS is associated with numerous comorbidities (269). If patients with ACS do not undergo surgery initially, they should be annually reassessed for cortisol hypersecretion and potential worsening of comorbidities. If there is clinical or biochemical progression then patients can be re-evaluated for surgery (341).

## The Future: Investigation and Management

Given the sensitivity and specificity limitations of standard endocrine testing (discussed in “**Endocrine Work-up of AI**”) and radiological investigations (discussed in “Imaging Evaluation of an AI”) in determining whether an adrenal lesion is benign or malignant, further advances are required to help risk-stratify patients with adrenal lesions.

### The role of urine steroid metabolomics

Standard serum steroid measurements are variable because of the diurnal variation in adrenal steroid secretion. Twenty-four-hour urine steroid excretion analysis represents a more accurate estimate of total corticosteroid production and of adrenal precursor steroids and metabolites (342, 343) and therefore has significant potential in the assessment of adrenal diseases. This has been extensively reviewed by Storbeck et al. (344).

Adrenal steroid production and metabolism are affected by sex, age, and BMI (342,345,346). Glucocorticoid metabolites are higher in men than in women and in obese than in lean persons (345,346). Assessing ratios of steroids that are substrates and products, respectively, of a distinct steroidogenic enzyme activity allows for representative assessment of elements of the steroidogenic pathway, which has been particularly informative in the diagnosis of inborn steroidogenic disorders, including CAH.

#### Mass spectrometry.

There are a number of different methods of mass spectrometry including gas chromatography-mass spectrometry (GC-MS) and LC-MS/MS (191,220,327), each of which has its pros and cons, which have been reviewed in detail by Krone et al. (347). In mass spectrometry, steroids are identified by unique retention times and or multiple reaction monitoring (206) with the area under the peak in the chromatogram is proportional to its concentration and quantifiable via comparison to standard reference materials of known concentration.

**Figure 9.**
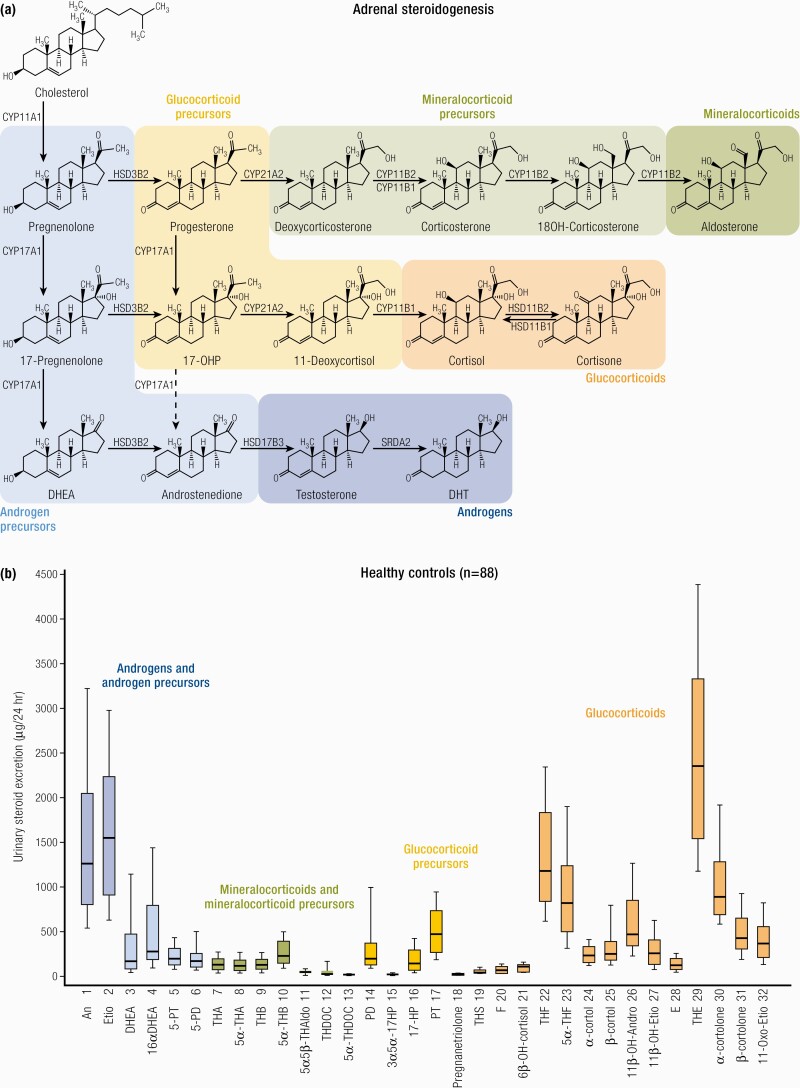
(A) Schematic representation of steroidogenesis depicting the major products of adrenocortical steroid synthesis, the mineralocorticoid aldosterone (dark green), and its precursors (light green), glucocorticoid precursors (yellow), the active glucocorticoid cortisol (orange) and its metabolite cortisone, and the adrenal androgens and their precursors (light blue). Synthesis of active androgens (dark blue) mainly takes place in the gonads. (B0. The 24-hour urinary steroid metabolite excretion in healthy controls (n=88). Box plots represent median and interquartile ranges; the whiskers represent 5th and 95th percentile, respectively. Color coding of steroid metabolites mirrors that used for depicting the major adrenal corticosteroid classes in (A). CYP, cytochrome P450; HSD, hydroxysteroid dehydrogenase; DHT, 5 α -dihydrotestosterone (342). From Arlt W, Biehl M, Taylor AE, Hahner S, Libe R, Hughes BA, Schneider P, Smith DJ, Stiekema H, Krone N, Porfiri E, Opocher G, Bertherat J, Mantero F, Allolio B, Terzolo M, Nightingale P, Shackleton CH, Bertagna X, Fassnacht M, Stewart PM. Urine steroid metabolomics as a biomarker tool for detecting malignancy in adrenal tumors. J Clin Endocrinol Metab 2011; 96:3775-3784 (Under Open Access License).

Urinary steroid profiling using GC-MS permits the analysis of multiple corticosteroids and their metabolites from a single urine sample (347) (Fig. 9). As most urinary steroids are conjugated, samples need to be deconjugated followed by a derivatization step before analysis which is both time consuming and labor intensive. LC-MS/MS is considerably faster and less labor intensive and allows a much higher throughput than GC-MS; however, LC-MS/MS has a poorer resolving capacity (206). As a result, LC-MS/MS is more suited to a smaller panel of corticosteroids and their metabolites and as a result does not have the same broad scope of analysis that GC-MS allows (206). Given the preanalytical and analytical advantages of LC-MS/MS over GC-MS, it is better suited to clinical practice once adequate optimization has been performed. However, GC-MS has several advantages, particularly in the research environment, over LC-MS/MS, including a better chromatographic resolution which permits the ready characterization of structures; use of GC-MS in scan mode allows for nontargeted steroid profiling and the discovery of novel compounds, metabolomes, and pathways of synthesis and metabolism. Importantly, scanned data can be stored indefinitely for future research (347).

#### Use of urinary steroid metabolome in research and clinical practice.

Increasingly, the results from panels of multiple steroids are being analyzed using novel statistical technical techniques including machine learning (which allows for the discovery of diagnostic and disease-specific patterns) revealing a personalized “urinary steroid metabolome.” This approach has been used for some time for diagnosing inborn errors of steroidogenesis such as CAH but has recently been developed for the evaluation of functionality and malignant potential of patients with adrenal nodules (281).

#### Use of urinary corticosteroid metabolism markers in differentiating between benign and malignant adrenal lesions.

**Figure 10.**
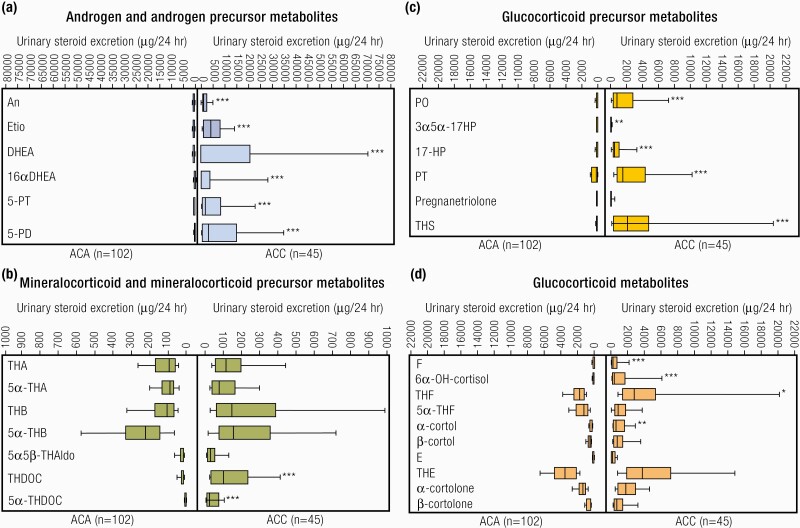
Steroid metabolite excretion in ACA (n = 102) and ACC (n = 45) according to steroid classes. (A) Metabolites of adrenal androgen precursors and active androgens; (B) metabolites of mineralocorticoids and their precursors; (C) metabolites of glucocorticoid precursors; (D) cortisol and cortisone metabolites. Box plots represent median and interquartile ranges; the whiskers represent 5th and 95th percentile, respectively.*P < .05; **P < .01; ***P < .001 comparing ACA with ACC. (342) From Arlt W, Biehl M, Taylor AE, Hahner S, Libe R, Hughes BA, Schneider P, Smith DJ, Stiekema H, Krone N, Porfiri E, Opocher G, Bertherat J, Mantero F, Allolio B, Terzolo M, Nightingale P, Shackleton CH, Bertagna X, Fassnacht M, Stewart PM. Urine steroid metabolomics as a biomarker tool for detecting malignancy in adrenal tumors. J Clin Endocrinol Metab 2011; 96:3775-3784 (Under Open Access License).

This area has been extensively reviewed recently by Bancos et al. (342,348-350). Studies have shown that ACCs have a distinct pattern of urinary corticosteroids, characterized by an excess of precursor steroid metabolites (Fig. 10 (342)). In particular, the cosecretion of a distinct combination of glucocorticoid and androgen metabolites is emerging as a sensitive diagnostic tool for ACC (342,348,349).

The study by Arlt et al. (342) assessed the 24-hour urinary steroid metabolome using GC-MS in 102 patients with benign adrenal adenomas and 45 patients with ACC. They used generalized matrix learning vector quantization to identify the most discriminate steroids which differentiated between a benign adenoma and a malignant ACC. They reported that 27% of patients with ACC had no hormonal excess on routine biochemical testing. Urinary steroid profiling revealed distinct differences between adrenocortical adenomas and ACC patients (Fig. 10). ACC patients had a significantly higher excretion of androgen precursor metabolites, metabolites of active androgens, deoxycorticosterone, and glucocorticoid precursor metabolites were significantly higher in ACC, as was free cortisol.

ACC patients exhibited significantly increased excretion of metabolites derived from the precursors of androgen, mineralocorticoid, and glucocorticoid synthesis. Even when analyzing the ACC patients without evidence of hormone excess on routine biochemistry, urinary excretion of androgen, and glucocorticoid precursor metabolites remained significantly increased. By contrast, steroid precursor metabolite excretion in nonmalignant AI did not differ from healthy controls. The majority of ACC patients (69%) showed androgen precursor excess, either combined with increased excretion of glucocorticoid precursors (36%) or both glucocorticoid and mineralocorticoid precursors (33%). There was no effect of age, sex, tumor size, or presence of metastasis on the characteristics of the urine steroid metabolomes in ACC and ACA patients. Using machine learning, the authors were able to identify that tetrahydro-11-deoxycortisol (THS), pregnenetriol (5-PT), and pregnenediol (5-PD) were the most discriminatory between ACC and ACA. They then included the next 6 most discriminatory to develop a panel that could be used for future validation on an LC/MS-MS platform (342).

Kotłowska et al. (343) have also reported the use of GC-MS analysis of 19 major urinary corticosteroids and their metabolites in 30 controls and 28 patients with “nonsecretory” incidentalomas. In this study, the diagnosis of an adrenal cancer was excluded by “examining the diameter of the tumor, its clinical image and properties revealed during additional test” and all adenomas were less than 4 cm as all lesions greater than this were surgically removed. Interestingly, 25% of patients with “nonsecretory” AIs had distinct patterns of urinary steroids compared to the controls and other patients with AIs. There is, therefore, the possibility that some of these patients could have had an autonomously secreting lesion or a small ACC.

In 2015, Kerkhofs et al. (348) reported data relating to 27 ACCs and 125 ACA patients. They performed GC-MS analysis of 22 steroid metabolites and identified 15 individual steroid markers with a sensitivity of 90% or above in detecting ACC, with the 11-deoxycortisol metabolite (THS) again representing the most informative marker. Other informative markers identified by that study included pregnanediol, pregnanetriol, etiocholanolone, the major androgen metabolite androsterone, and the major glucocorticoid metabolites tetrahydrocortisol and tetrahydrocortisone. In a recent study by Velikanova et al. (349), assessing 32 steroid metabolites by GC-MS in 24-hour urine samples from 31 patients with ACC and 96 patients with ACA, the authors confirmed the findings reported by Arlt et al. (342). Similar to Kerkhofs et al. (348) they did not employ unbiased computational data analysis of the steroid excretion data. All studies identified THS as the most informative steroid marker indicative of ACC. THS is the metabolite of 11-deoxycortisol, which is converted to cortisol by the adrenal-specific steroidogenic enzyme cytochrome P450 family 11 subfamily B member 1 (*CYP11B1*). Measurement of 11-deoxycortisol is not routinely used clinically, though recently serum results were reported in a cohort of benign adrenal tumors (249).

A recent study has also shown the utility of urinary steroid metabolomics in the follow up of patients with ACC as a marker of recurrence (351).

#### Future directions and challenges with steroid metabolomics.

Arlt et al. have used GC-MS analysis to reveal previously unrecognized significant glucocorticoid excess in patients with primary hyperaldosteronism, labeled “Connshing” syndrome (281). They performed mass spectrometry-based analysis (GC-MS) of a 24-hour urine steroid metabolome in patients with primary aldosteronism in comparison to healthy controls, patients with endocrine inactive adrenal adenoma, patients with ACS, and with clinically overt adrenal cortisol excess. Patients with primary hyperaldosteronism had significantly increased cortisol and total glucocorticoid metabolite excretion. Several surrogate parameters of metabolic risk (including BMI, waist–hip ratio, fasting insulin, HOMA-IR, and high-density lipoprotein cholesterol) correlated significantly with glucocorticoid but not mineralocorticoid output.

Unilateral adrenalectomy resolved both mineralocorticoid and glucocorticoid excess, indeed, postoperative evidence of adrenal insufficiency as evidenced by a suboptimal cortisol response to synacthen testing was found in 29% of patients despite a normal preoperative dexamethasone suppression test.

These data are of significant interest given the other comorbidities reported in patients with primary hyperaldosteronism such as increased rates of insulin resistance/type 2 diabetes and osteoporosis, which were difficult to attribute to aldosterone effect per se (281). More research is needed in this area as it raises several important questions, not least the impact of this glucocorticoid excess in patients who are not surgically cured of their primary hyperaldosteronism but receive mineralocorticoid receptor antagonist therapy which has no antiglucocorticoid effect. Further prospective data are required to validate these retrospective studies.

There are several important future directions of research in this area including the potential to use a spot urine sample rather than a 24-hour collection and transferring this technology to an LC/MS-MS based high throughput platform which will be easily performed in routine biochemistry laboratories. In recent years, LC-MS/MS has also been used on serum samples to differentiate between benign and malignant adrenal disease (352,353).

Ultimately, the use of a noninvasive steroid metabolomic assessment may be able to risk-stratify patients rapidly and accurately, thus leading to prompt and appropriate therapy for patients with ACC and a reduction in the need for follow-up of patients with benign adrenal disease and unnecessary surgery for patients with radiologically indeterminate lesions. The combination of the above will hopefully lead to improved survival for patients with ACC as they are recognized and treated promptly and a decrease in healthcare costs (due to a reduction in surgical procedures) and exposure to ionizing radiation by reducing CT imaging in patients with benign disease (45).

### Other diagnostic innovations

There are a number of other exciting developments in the field of adrenal tumors in terms of improved histopathological techniques that include In situ metabolomics utilizing high‑mass resolution matrix‑assisted laser desorption/ionization mass spectrometry imaging, which has been extensively reviewed by Papathomas et al. (206) and is beyond the scope of this review. Other advances include the use of molecular biology techniques to predict prognosis in patients with ACC (165,354-356) which may give the promise of delivering a more personalized approach to prognostication, treatment, and follow-up.

#### Functional imaging of the adrenal cortex.

Scintigraphy techniques have been used for imaging of the adrenal glands for several decades. iodine-131 labelled 6-beta-iodomethyl-19-norcholesterol (NP-59), a cholesterol analog that incorporates into low-density lipoproteins and accumulates in the adrenal cortex via a receptor-mediated process, has been used for imaging of the adrenal cortex as a marker of adrenal cholesterol metabolism since the 1970s (357). The emergence of integrated single-photon emission computed tomography-computed tomography (SPECT-CT) systems has led to a resurgence of interest in the use of NP-59 scintigraphy (358). Recent data have reported a positive predictive value of 92% for differentiation of aldosterone-producing adenoma from bilateral adrenal hyperplasia in primary aldosteronism, potentially reducing the need for invasive adrenal venous sampling (359). Limitations of NP-59 scintigraphy including the requirement for prolonged dexamethasone suppression, the need for sequential imaging over several days, and relatively high radiation dose, have stimulated the development of other functional imaging techniques in the diagnosis of primary aldosteronism (360).

**Figure 11.**
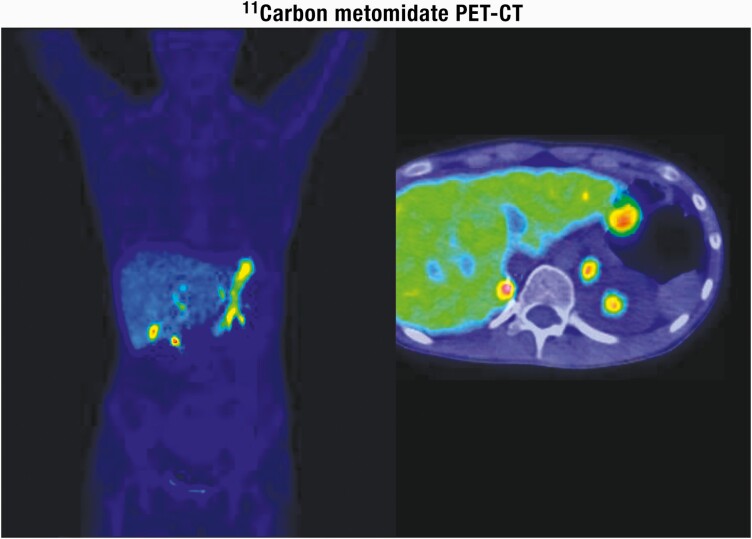
Carbon-11 metomidate PET-CT: Coronal PET maximum intensity projection and axial fused PET-CT images in a patient with a left adrenocortical carcinoma and an unsuspected tracer-avid right sided liver metastasis. Prominent physiological tracer uptake is present in the gastric wall and left renal collecting system. Images courtesy of Professor Anders Sundin, Consultant Radiologist, Uppsala University Hospital, Sweden.

Etomidate derivatives with high affinity and specificity for enzymes within the cytochrome P450 11B family (*CYP11B*) which are expressed exclusively within adrenocortical cells have been developed (361). *CYP11B1* (11-beta-hydroxylase) and *CYP11B2* (aldosterone synthetase) enzymes are key regulators of adrenal cortisol and aldosterone synthesis (362). Carbon-11 (^11^C) metomidate (MTO) PET-CT is a sensitive and specific technique for diagnosing adrenocortical tumors (363) and has high accuracy for lateralizing aldosterone secretion by Conn’s adenomas (364,365). The 20-minute half-life of ^11^C restricts the use of this tracer to centers with an on-site cyclotron and consequently, this technique is not widely available (Fig. 11). This limitation has led to the development of longer half-life radiotracers which are required before more widespread availability can be achieved. Recently, an ^18^F-labeled analog of MTO (^18^F-FAMTO) has been developed with high selectivity for CYP11B enzymes (366). ^123^I-labeled iodometomidate (IMTO) has also been evaluated as a potentially more widely available radiotracer (367). ^123^I-IMTO SPECT-CT permits highly specific characterization of adrenocortical tissue with lower radiation exposure and investigation than NP-59 scintigraphy (368) and also has reported value for the staging of ACC (369). Labeling of metomidate with iodine-131 offers the potential for targeted radionuclide therapy in advanced adrenocortical cancer but this requires validation in a prospective clinical trial (370).

#### Functional imaging of the adrenal medulla.

**Figure 12.**
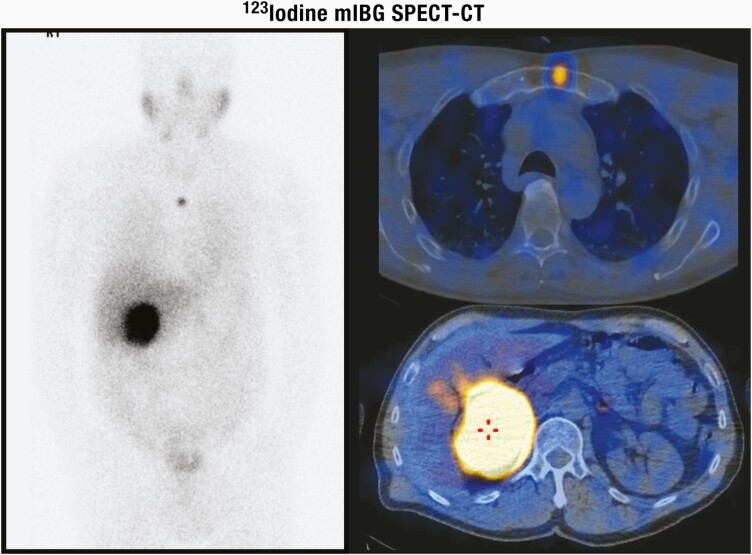
Iodine-123 metaiodobenzylguanidine SPECT-CT. Anterior half-body planar view and axial fused SPECT-CT images in a patient with a large MIBG-avid right adrenal pheochromocytoma with an unsuspected tracer-avid bone metastasis within the manubrium.

**Figure 13.**
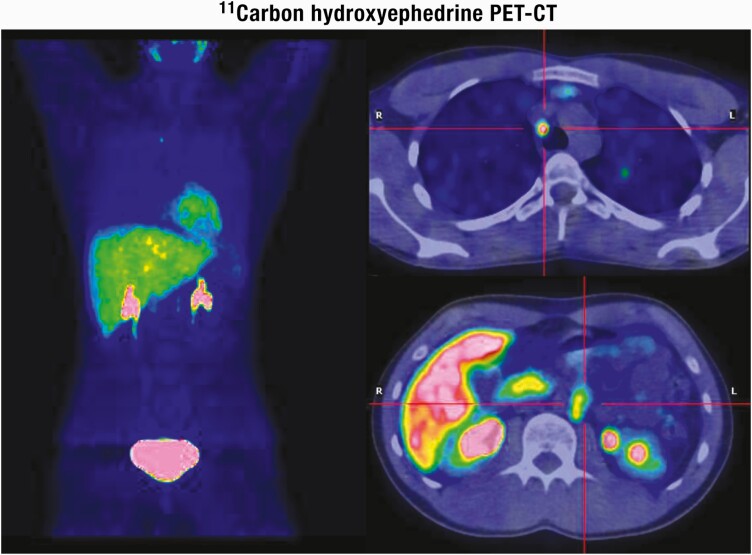
Carbon-11 hydroxyephedrine PET-CT. Coronal PET maximum intensity projection and axial fused PET-CT images in a patient with a left adrenal pheochromocytoma and an unsuspected tracer-avid subcentimeter right paratracheal node which was biopsied and confirmed to represent a nodal metastasis. Images courtesy of Professor Anders Sundin, Consultant Radiologist, Uppsala University Hospital, Sweden.

Iodine-labelled metaiodobenzylguanidine (MIBG), a guanethidine analog, has been used for functional imaging of tumors arising from the adrenal medulla for over 30 years (371). ^123^I-MIBG has significant limitations, including a 2-day imaging protocol and the need for thyroid blockade with potassium iodide prior to tracer injection (Fig. 12). ^124^I-MIBG has been evaluated as a potential PET imaging tracer but this undergoes complex decay with emission of high energy gamma radiation leading to poorer image quality and unfavorable dosimetry (372). Other PET tracers including dopamine analogs such as ^18^F-fluorodopamine and ^18^F-fluorodihydroxyphenylalanine (DOPA) which image norepinephrine (noradrenaline) and amino acid transporter expression, along with ^11^C-hydroxyephedrine, a catecholamine analog, have been used to evaluate tumors arising from the neural crest (373) (Fig. 13). These tracers are limited to highly specialized centers and the published data is limited to relatively small cohorts. Recently an ^18^F-labeled analog of MIBG has been developed, meta-fluorobenzylguanidine (^18^F-MFBG); preliminary data report high accuracy and favorable dosimetry in a single-day protocol overcoming some of the limitations of other tracers and showing promise for the future (374).

### Textural analysis/radiomics

**Figure 14.**
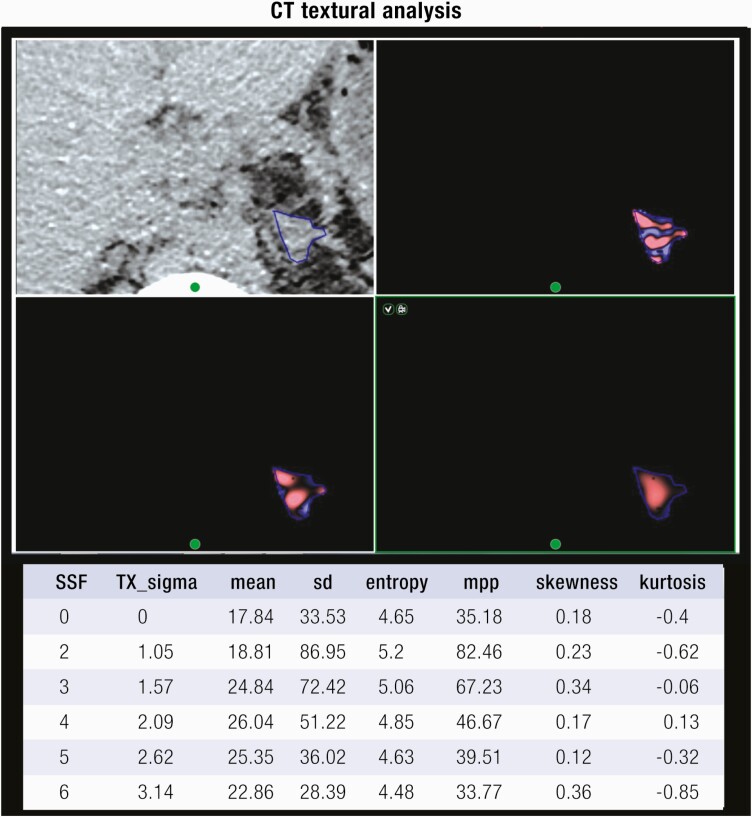
CT textural analysis. Axial CT image showing left adrenal lesion segmentation (blue contours, top left) and adrenal lesion textural features displayed using fine (top right), medium (bottom left) and coarse (bottom right) filters using TexRADTM software. First-order textural parameters extracted from the lesion are tabulated below. SSF, spacial scaling factor; SD, standard deviation; MPP, mean of positive pixels.

Radiomics is a relatively new area of research activity which involves the extraction of “invisible” quantitative features from medical imaging based on intensity, shape, volume, and textural features (375). Textural analysis permits the evaluation of spatial inter-relationships within lesions to quantify lesion heterogeneity (376). The potential to obtain additional data from standard-of-care imaging could improve diagnostic accuracy and help guide optimal patient management (377) (Fig. 14).

Preliminary data on the use of CT textural analysis for the characterization of indeterminate adrenal lesions has shown promising results. In a feasibility study of 164 patients with indeterminate adrenal lesions at CT (98 pheochromocytoma; 66 lipid-poor adrenal adenomas), intralesion textural features (mean gray-level intensity and mean positive pixels) extracted from unenhanced images allowed differentiation between adenoma and pheochromocytoma with an accuracy of 81% (378). In a recent larger patient cohort (n = 225) the same Chinese group has reported an accuracy of 77% for distinguishing metastases from benign adrenal lesions using this CT textural feature analysis technique (379). Other groups have shown similar results in small retrospective series evaluating the accuracy of MRI and PET-CT textural features for distinguishing benign and malignant adrenal lesions (380,381).

### Machine learning and imaging

There is a great interest in the use of artificial intelligence and machine learning techniques in medical imaging (382). The combination of textural analysis with machine learning classification may improve diagnostic accuracy and reproducibility. Early data evaluating the efficacy of textural analysis-derived parameters extracted from MRI of adrenal lesions in 60 patients using a machine learning approach showed a diagnostic accuracy of 80% compared to expert radiologist accuracy of 73% (383). A feasibility study assessing machine learning-based quantitative textural analysis of unenhanced CT in 108 patients with indeterminate adrenal lesions had a 94% accuracy for differentiating pheochromocytoma from lipid-poor adenoma (384). At present, there is insufficient evidence to support clinical translation, but these techniques have the potential to improve diagnostic accuracy and more informed decision making in the future. These initial findings warrant validation in a well-designed prospective multicenter clinical trial.

## Conclusion

An AI is now established as a common endocrine diagnosis that requires a multidisciplinary approach for effective management. The majority of patients can be reassured and discharged, but a personalized approach based upon image analysis, endocrine workup, and clinical symptoms and signs is required in every case. ACC remains a real concern but is restricted to <2% of all cases. Functional AI lesions are commoner (but still probably <10% of total) and the greatest challenge remains the diagnosis and optimum management of ACS. Modern-day surgery has improved outcomes and novel radiological and urinary biomarkers will improve early detection and patient stratification in future years to come.

## Data Availability

All data generated or analyzed during this study are included in this published article or in the data repositories listed in References.

## Abbreviations

17-OHP: 17-hydroxyprogesterone
ACA: adrenocortical adenoma
ACC: adrenocortical carcinoma
ACS: autonomous cortisol secretion
ACTH: adrenocorticotropin
AI: adrenal incidentaloma
BMI: body mass index
CAH: congenital adrenal hyperplasia
cAMP: 3′,5′-cyclic adenosine 5′-monohosphate
COMT: catechol-O-methyltransferase
CT: computed tomography
DHEA: dehydroepiandrosterone
DHEAS: DHEA sulfate
FDG: 18-fluorodeoxyglucose
GC-MS: gas chromatography-mass spectrometry
HPA: hypothalamic–pituitary–adrenal
HU: Hounsfield units
LC-MS/MS: liquid chromatography tandem mass spectrometry
MACE: mild autonomous cortisol excess
MEN: multiple endocrine neoplasia
MRI: magnetic resonance imaging
MTO: metomidate
NF1: neurofibromatosis type 1
NFAT: nonfunctioning adrenal tumor
PA: primary aldosteronism
PET: positron emission tomography
PKA: protein kinase A
PBMAH: primary bilateral macronodular adrenal hyperplasia
SPECT-CT: single-photon emission computed tomography-computed tomography
SUV: standardized uptake value
TH: tyrosine hydroxylase
THS: tetrahydro-11-deoxycortisol
UFC: urine free cortisol
VHL: Von Hippel Lindau

## Additional Information

***Disclosure Summary:*** All authors have submitted the ICMJE Form for Disclosure of Potential Conflicts of Interest. Conflicts that the editors consider relevant to the content of the manuscript have been disclosed. I certify that neither I nor my co-authors have a conflict of interest as described above that is relevant to the subject matter or materials included in this Work.
